# Indoleamine 2,3-dioxygenase 1 limits hepatic inflammatory cells recruitment and promotes bile duct ligation-induced liver fibrosis

**DOI:** 10.1038/s41419-020-03277-0

**Published:** 2021-01-07

**Authors:** Chan Mo, Shuwen Xie, Bin Liu, Weichao Zhong, Ting Zeng, Sha Huang, Yuqi Lai, Guanghui Deng, Chuying Zhou, Weixin Yan, Yuyao Chen, Shaohui Huang, Lei Gao, Zhiping Lv

**Affiliations:** 1 School of Traditional Chinese Medicine, Southern Medical University, 510515 Guangzhou, Guangdong People’s Republic of China; 2 Department of Emergency, Guangzhou Red Cross Hospital, Medical College, Jinan University, 510220 Guangzhou, China; 3 Shenzhen Traditional Chinese Medicine Hospital, No.1, Fuhua Road, Futian District, 518033 Shenzhen, Guangdong People’s Republic of China; 4 The Key Laboratory of Molecular Biology, State Administration of Traditional Chinese Medicine, School of Traditional Chinese Medicine, Southern Medical University, 510515 Guangzhou, Guangdong People’s Republic of China; 5 Guangdong Provincial Key Laboratory of Shock and Microcirculation, Southern Medical University, 510515 Guangzhou, People’s Republic of China

**Keywords:** Differentiation, Antigen-presenting cells

## Abstract

Liver fibrosis is a course of chronic liver dysfunction, can develop into cirrhosis and hepatocellular carcinoma. Inflammatory insult owing to pathogenic factors plays a crucial role in the pathogenesis of liver fibrosis. Indoleamine 2,3-dioxygenase 1 (IDO1) can affect the infiltration of immune cells in many pathology processes of diseases, but its role in liver fibrosis has not been elucidated completely. Here, the markedly elevated protein IDO1 in livers was identified, and dendritic cells (DCs) immune-phenotypes were significantly altered after BDL challenge. A distinct hepatic population of CD11c^+^DCs was decreased and presented an immature immune-phenotype, reflected by lower expression levels of co-stimulatory molecules (CD40, MHCII). Frequencies of CD11c^+^CD80^+^, CD11c^+^CD86^+^, CD11c^+^MHCII^+^, and CD11c^+^CD40^+^ cells in splenic leukocytes were reduced significantly. Notably, IDO1 overexpression inhibited hepatic, splenic CD11c^+^DCs maturation, mature DCs-mediated T-cell proliferation and worsened liver fibrosis, whereas above pathological phenomena were reversed in IDO1^−/−^ mice. Our data demonstrate that IDO1 affects the process of immune cells recruitment via inhibiting DCs maturation and subsequent T cells proliferation, resulting in the promotion of hepatic fibrosis. Thus, amelioration of immune responses in hepatic and splenic microenvironment by targeting IDO1 might be essential for the therapeutic effects on liver fibrosis.

## Introduction

Liver fibrosis is a continuous chronic wound healing response to persistent hepatocyte injury, which develops secondary to infection, toxin, viral hepatitis, alcoholic liver diseases, cholestatic diseases, or immune-mediated attack etc.^1,2^. The activation of hepatic stellate cells (HSCs), which produce excessive extracellular matrixes and lead to an imbalance of collagen deposition and degradation, is the main pathological characteristics of liver fibrosis^3,4^. Hepatic fibrosis, which is an early stage of liver cirrhosis, may progress to hepatocellular carcinoma^5^. Approximately 2 million deaths worldwide due to liver disease, half of them owing to complications of cirrhosis, the other half due to viral hepatitis and hepatocellular carcinoma. Cirrhosis is the primary cause of disability-adjusted life years and years of life lost, which accounts for 1.6% and 2.1% of the worldwide burden^6^. Consequently, investigate the underlying mechanisms of hepatic fibrosis is of great importance.

Recently, the abnormal changes of hepatic immune microenvironment during hepatic fibrosis attracted increasing attention among foreign and domestic scholars^7–10^. Indoleamine 2,3-dioxygenase 1 (IDO1), which serves as an immunomodulatory molecule, is an intracellular enzyme that participates in the metabolism of the essential amino acid tryptophan (TRP) in the kynurenine (KYN) pathway^11–13^. A relevant study figured out that knock out of IDO1 reduced Th17 cells and further protected mice from CCL4-induced liver fibrosis^14^. IDO1 was dramatically upregulated during concanavalin A-induced acute immune hepatitis and inhibited of IDO1 could alleviate murine hepatic damage^15^. Several lines of evidence suggest that IDO1 is involved in regulating several kinds of immune cells. The expression level of IDO1 was related to FoxP3^+^Treg and CD8^+^T-cell infiltration during prostate cancer^16^. A novel negative regulator of IDO1, H2S, showed antitumor immunotherapeutic effects, including induced T-effector cells and inhibited myeloid-derived suppressor cells (MDSCs)^17^. Moreover, immune cells were required for the sustained IDO1 expression in human hepatoma cell lines^18^. Although the critical role of IDO1 participated in the process of immune regulation during multiple courses of diseases had been demonstrated, the distinct function of IDO1 in the change of immune responses during liver fibrosis has not been studied.

Dendritic cells (DCs), serve as professional antigen-presenting cells (APC), play an important part in capturing and presenting antigens to T cells^19,20^. Previous studies showed that DCs could limit fibro-inflammatory injury in non-alcoholic steatohepatitis and DCs contributed to CCL4-induced liver fibrosis regression^21,22^. Increasing research indicated that IDO1 was associated with the differentiation, maturation and function of DCs. IDO1 inhibitors had the capacity to enhance the stimulatory capacity of DCs^23^. JNK^+^IDO1^+^ BMDCs decreased the expression levels of MHC class II, CD80 and failed to stimulate allogeneic T cells^24^. IDO channel signaling may be involved in immature DC-induced allograft immunotolerance and associated with the suppression of T-cell responses^25^. However, whether IDO1 is involved in DCs activation and maturation during liver fibrosis and the underlying immunoregulatory mechanisms are still poorly understood.

The present work investigated the in vivo role of IDO1 in the pathogenesis of liver fibrosis through assessing the degree of liver fibrosis and maturation status of DCs induced by bile duct ligation (BDL) in wild-type (WT) mice, mice deficient in IDO1 and mice overexpression in IDO1. Our results suggested that IDO1 accelerated the development of BDL-induced liver fibrosis by altered hepatic inflammatory cells recruitment including reduced hepatic CD11c^+^DC populations, made DCs presented an immature phenotype and reduced subsequent T cells proliferation.

## Results

### IDO1 expression level was enhanced in fibrotic livers induced by BDL

**Fig. 1:**
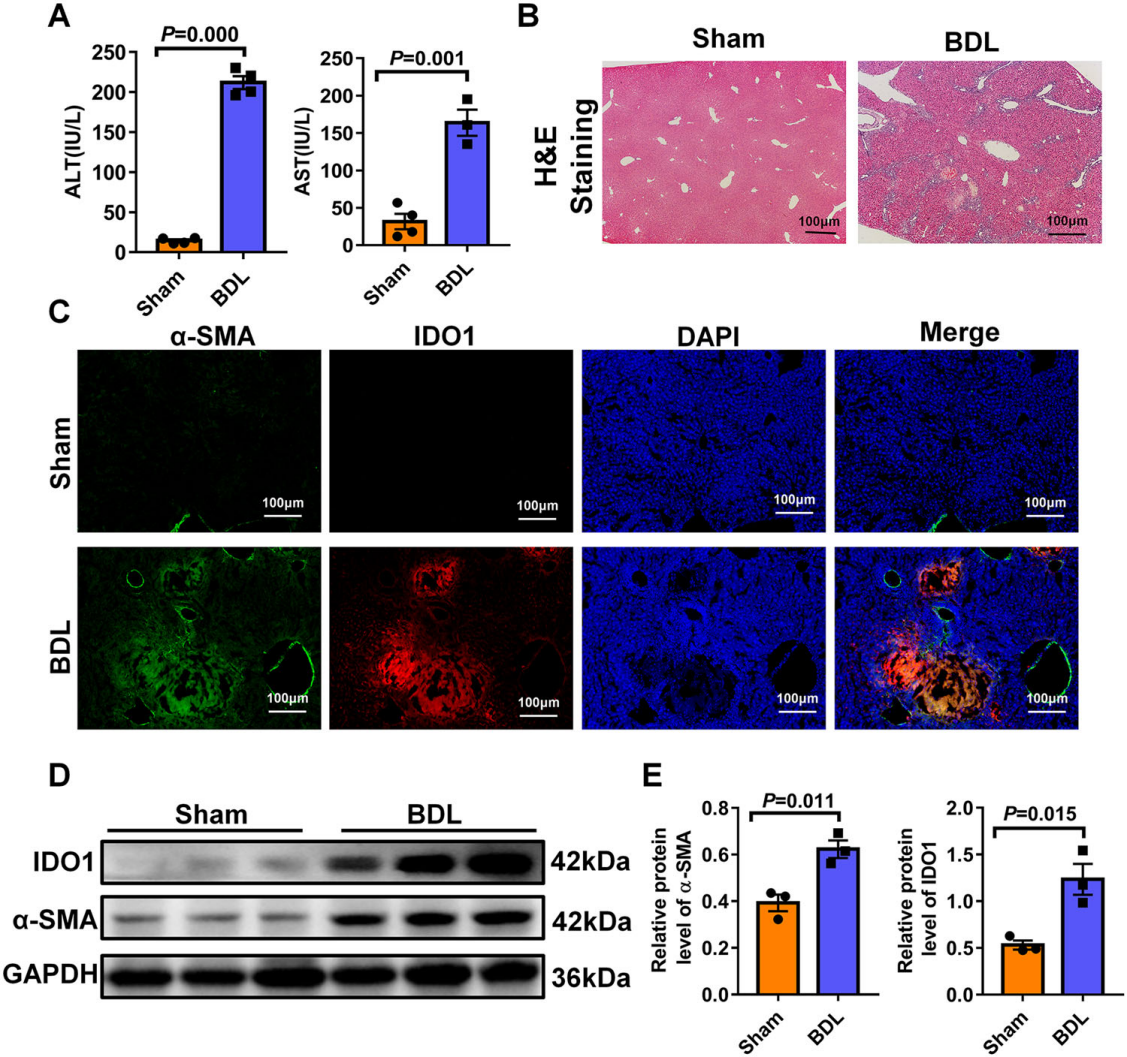
IDO1 expression level was enhanced in fibrotic livers induced by BDL. **A** Serum concentrations of AST and ALT were measured from sham, BDL-operated mice (*n* ≥ 3). **B** H&E staining of liver sections. **C** Liver samples were processed for immunofluorescence co-staining for IDO1 with α-SMA. Slides were counterstained with DAPI. **D** Western blot analysis of the expression of IDO1 and α-SMA in the livers of WT control and model mice (*n* = 3). **E** GAPDH served as a loading control. The density of GAPDH, α-SMA and IDO1 protein in **D** were measured using image J software and quantified in a bar graph, respectively. In all panels, scale bars, 100 mm. The data are presented as the means ± SEM. *P* < 0.05 is considered statistically significant. Statistical analysis was analyzed by independent-samples *t-*test.

To investigate the pathological role of IDO1 in liver fibrosis, we found the classical animal model of liver fibrosis by bile duct ligation. In response to bile duct ligation, mice exhibited significantly increased levels of ALT and AST as compared with sham operation group (Fig. 1A). Histologic examination of liver sections stained with hematoxylin eosin (H&E) indicated bile duct ligation led to the necrosis of hepatocytes, collagen deposited around the central veins and the formation of pseudo-lobule (Fig. 1B). The activation of hepatic stellate cells (HSCs) was a key pathological process in the development of fibrogenesis, which was characterized by an abnormal increased expression level of α-SMA. As showed in Fig. 1C, the model mice had higher α-SMA expression level and more α-SMA-positive cells in their liver tissues than the normal mice. Importantly, in the normal livers, a low expression level of IDO1 was observed, whereas in the fibrotic livers, significantly stronger IDO1 expression was detected in the areas surrounding portal vessels, damaging boundaries. Furthermore, IDO1 expression was overlapped with α-SMA-positive HSCs as evaluated by immunofluorescence co-staining for IDO1 and α-SMA (Fig. 1C). Western blot analysis also revealed that fibrotic liver samples exhibited much higher protein levels of α-SMA as well as IDO1 than the normal livers (Fig. 1D, E). The above findings suggest that IDO1 was enhanced with activated HSCs in fibrotic liver, and therefore may be related to liver fibrosis.

### Hepatic and splenic CD11c^+^DC populations were decreased in BDL-induced liver fibrosis

**Fig. 2:**
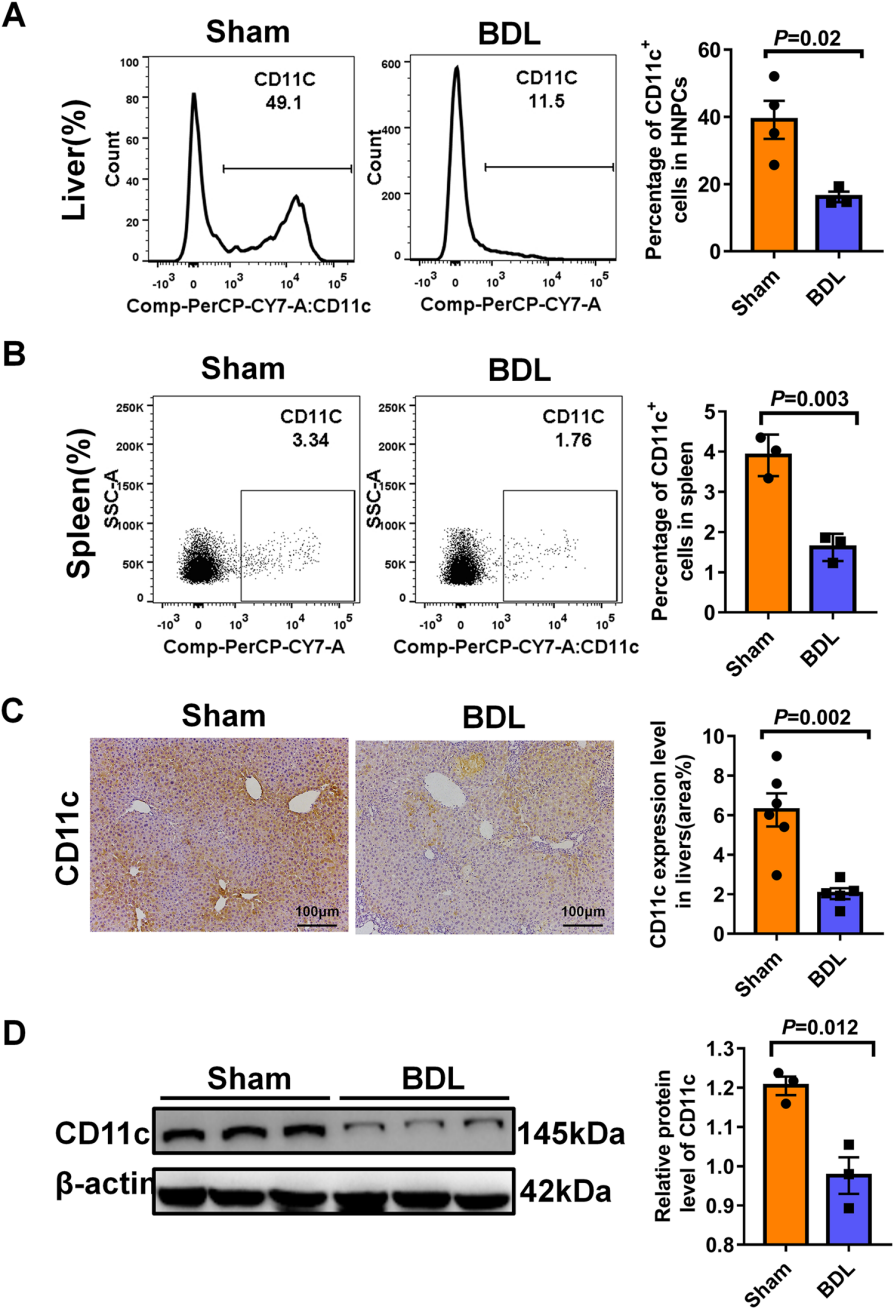
Hepatic and splenic CD11c^+^DC populations were decreased in BDL-induced liver fibrosis. **A** Representative FACS plots (Left) and summary data (right) showing the percentage of hepatic CD11c^+^DC cells in hepatic NPCs (*n* ≥ 3). **B** Representative FACS plots (left) and summary data (Right) showing the percentage of splenic CD11c^+^DC cells in spleen leukocytes (*n* = 3). **C** Immunohistochemistry analysis to detect the expression of CD11c in the livers of sham, BDL-operated mice. The area of CD11c staining was measured using image J software (*n* = 5–6). **D** Western blot analysis of the expression of CD11c in the livers of WT control and model mice. In all panels, scale bars, 100 mm. The data are presented as the means ± SEM. *P* < 0.05 is considered statistically significant. Statistical analysis was analyzed by independent-samples *t-*test.

It is known that hepatic DCs could induce either immunogenic responses or tolerance according to physiologic circumstances and affect disease course^26^. Hepatic non-parenchymal cells(HNPCs) isolated from bile duct ligation or sham-operated mice were analyzed for DCs frequencies by flow cytometry. As shown in Fig. 2a, hepatic CD11c^+^DCs lessened from ~39.1% in controls to ~16.2% in BDLs. Consistently, we also observed fewer CD11c-positive cells in the fibrotic livers compared to normal livers, as detected by CD11c immunohistochemistry staining (Fig. 2C). Western blot analysis also revealed that BDL-operated reduced hepatic CD11c protein levels as compared to sham-operated (Fig. 2D). Moreover, splenic leukocytes harvested from BDL-operated mice exhibited a significant decrease in the frequency of CD11c^+^ DC cells, implying that the effects of BDL on DCs are specific to both liver and spleen (Fig. 2B).

### Hepatic and splenic DCs exhibited an immature phenotype in BDL-induced liver fibrosis

**Fig. 3:**
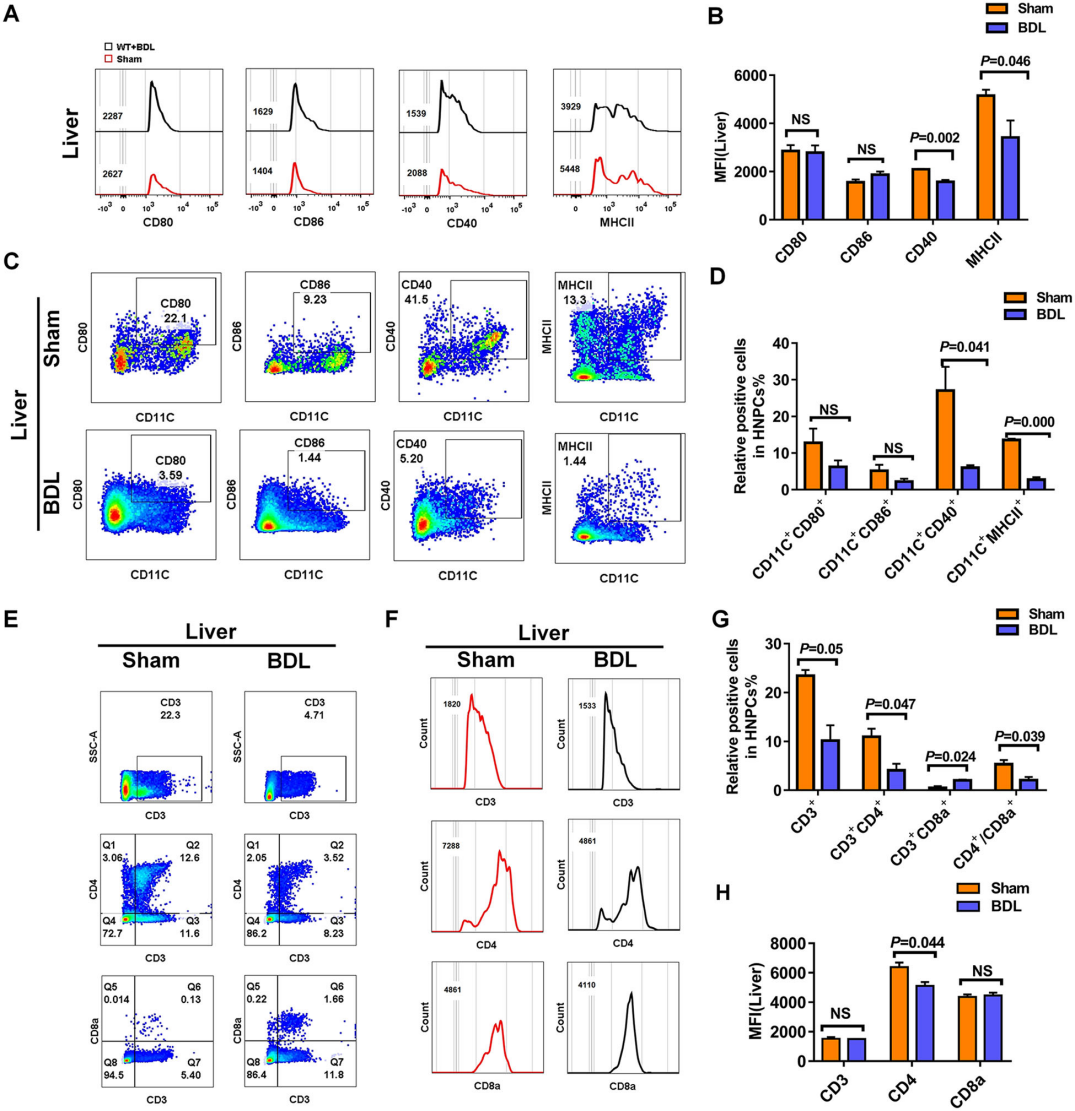
Hepatic and splenic DCs exhibited an immature phenotype in BDL-induced liver fibrosis. **A** Activation marker expression on hepatic DCs in sham, operated mice were measured by flow cytometry and summarized data were listed on right (**B**) (*n* ≥ 3). **C** Representative FACS plots showing CD11c^+^CD80^+^, CD11c^+^CD86^+^, CD11c^+^MHCII^+^, and CD11c^+^CD40^+^ cells in WT control, WT model mice and summarized data were listed on right (**D**) (*n* ≥ 3). **E** Frequencies of CD3^+^, CD3^+^CD4^+^, CD3^+^CD8^+^ T cells in hepatic NPCs were assessed by flow cytometry and summarized data were listed on right (**G**) (*n* ≥ 3). **F** MFI of CD3, CD4, CD8a in hepatic NPCs were measured by flow cytometry and summarized data were listed on right (**H**) (*n* ≥ 3). The data are presented as the means ± SEM. *P* < 0.05 is considered statistically significant. Statistical analysis was analyzed by independent-samples *t*-test.

Besides lessening in number, hepatic DCs underwent phenotypic changes in liver fibrosis induced by BDL. We observed a significant decrease in the frequencies of CD11c^+^MHCII^+^, CD11c^+^CD40^+^ cells in hepatic NPCs from BDL-operated mice compared to those from sham-operated mice (Fig. 3C, D). Furthermore, the expression levels of activation markers MHCII and CD40, both crucial for antigen presentation, were downregulated on hepatic CD11c^+^ DCs after BDL-operated, as reflected by a significant decrease in mean fluorescence intensity (MFI) of MHCII, CD40 (Fig. 3A, B). However, the expression levels of co-stimulatory molecules CD80, and CD86 on hepatic CD11c^+^ DCs did not differ between hepatic NPCs obtained from fibrotic and normal mice, BDL-operated had no influence on CD11c^+^CD80^+^, CD11c^+^CD86^+^ populations in hepatic NPCs (Fig. 3A–D).

According to the decreased numbers of hepatic CD11c^+^DCs and lower expression levels of MHCII and CD40 on hepatic CD11c^+^DCs in BDL-operated mice, we suspected that the activation and differentiation of hepatic T-cells might be affected, as well. As respected, a robust reduction in the number of hepatic CD3^+^, CD3^+^CD4^+^ T cells were observed in BDL-operated mice as compared with sham-operated controls, whereas the frequency of hepatic CD3^+^CD8^+^ T cells exhibited a reverse changing direction after BDL-operated. Moreover, hepatic T cells in BDL-operated mice also had a lower ratio of CD4^+^/CD8^+^ compared with sham-operated mice (Fig. 3E, G).

We have previously found that the splenic CD11c^+^DC population was decreased in BDL-induced liver fibrosis. Splenic leukocytes of BDL and sham-operated mice were further analyzed for CD11c^+^DC surface markers expression by flow cytometry. We detected a significant decrease in the percentage of CD11c^+^CD80^+^, CD11c^+^CD86^+^, CD11c^+^MHCII^+^ and CD11c^+^CD40^+^ cells in splenic leukocytes from BDL-operated mice compared to those from sham-operated controls. Splenic leukocytes displayed no difference in the MFI of cell surface co-stimulatory molecules (MHCII, CD40, CD80, CD86) on CD11c^+^DCs between BDL-operated and sham-operated mice (Fig. S1A–C). However, the frequencies of splenic CD3^+^, CD3^+^CD4^+^ T cells did not vary between BDL-operated and sham-operated mice. Moreover, splenic leukocytes harvested from BDL-operated mice exhibited a higher frequency of CD3^+^CD8^+^ T cells compared to sham-operated controls, whereas the ratio of splenic CD4^+^/CD8^+^ was reduced in BDL-operated mice (Fig. S2A–C). We failed to detect any difference between BDL-operated and sham-operated mice in the MFI of splenic CD3, CD4 and CD8a (Fig. S2D, E). Additionally, the frequency of CD3^+^ T cells in the thymus of BDL-operated mice showed a strong increased compared to sham-operated controls, whereas BDL-operated mice had a lower frequency of splenic CD3^+^CD4^+^ T cells as well as splenic CD4^+^/CD8^+^ compared to sham-operated controls (Fig. S3A–C).

Taken together, these data indicated that, in liver fibrosis induced by BDL, both hepatic and splenic CD11c^+^DCs underwent a change in maturation and alteration in subset composition. The capacity of hepatic CD11c^+^DCs in BDL-operated mice to induce CD3^+^, CD3^+^CD4^+^ T cells proliferation were decreased, whereas both hepatic and splenic CD11c^+^DCs gained enhanced capacity to activate CD3^+^CD8^+^ T cells and resulted in a decreased ratio of CD4^+^/CD8^+^. Similarly, the trend of changes in the frequency of thymic CD3^+^CD4^+^ T cells and the ratio of CD4^+^/CD8^+^ in BDL-operated mice were same to hepatic NPCs. However, the frequency of CD3^+^ T cells appeared a compensatory increase in thymic leukocytes. These data suggest that downregulation of activation markers on CD11c^+^DCs and possibly aberrant suppression of subsequent T cells may contribute to the development of liver fibrosis.

### Knock out of IDO1 ameliorated the development of BDL-induced liver fibrosis in mice

**Fig. 4:**
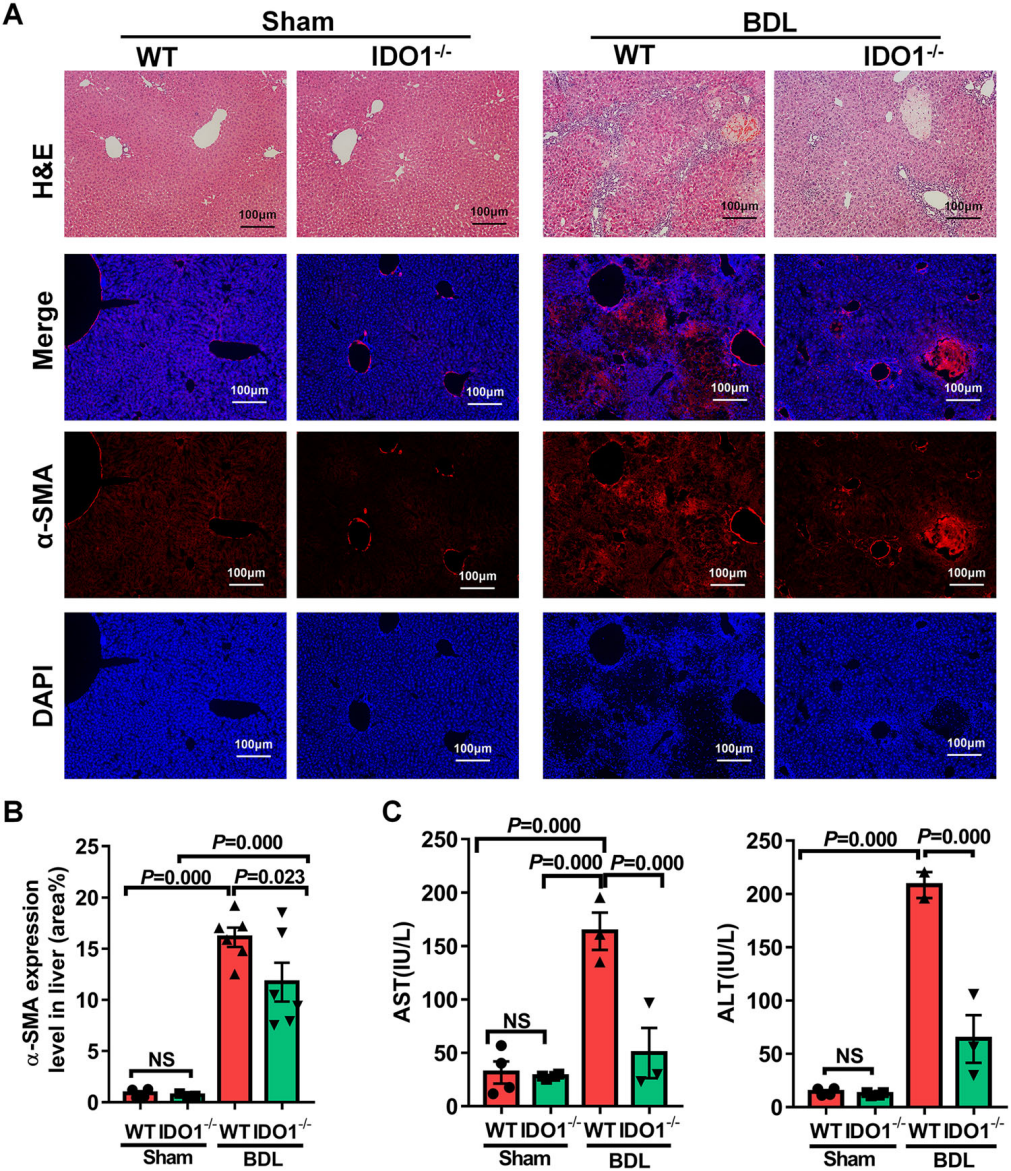
Knock out of IDO1 ameliorated the development of BDL-induced liver fibrosis in mice. **A** H&E and α-SMA immunofluorescence staining of liver sections in WT and IDO1^–/–^ mice. **B** α-SMA quantification of positive-stained areas was shown in the graph (*n* ≥ 3). **C** Liver injury was assessed by measuring serum ALT and AST levels in WT and IDO1^–/–^mice (*n* ≥ 3). In all panels, scale bars, 100 mm. The data are presented as the means ± SEM. *P* < 0.05 is considered statistically significant. Statistical analysis was analyzed by one-way ANOVA.

After founding the accumulation of IDO1 in the liver of BDL-operated mice, we next employed IDO1^−/−^ mice to confirm whether IDO1 regulates liver fibrogenesis. The genotype of IDO1^−/−^ mice were identified according to the standard PCR protocols posted on the Jackson Laboratory’s official website (Fig. S4). As presented in Fig. 4, in the absence of damage, there were no significant differences in the serum levels of AST and ALT, as well as histologic examination of liver sections stained with H&E and α-SMA immunofluorescence staining between WT and IDO1^−/−^ mice. These results suggested that knock out of IDO1 in mice might not induce liver fibrosis spontaneously in the absence of damage. Consequently, IDO1^−/−^ mice were challenged with BDL lasted for 3 weeks to evaluate the effects of IDO1 gene deletion to the outcome of liver fibrosis. IDO1^−/−^ fibrotic mice, in comparison to WT fibrotic mice, exhibited reduced inflammatory infiltrates around affected bile ducts, decreased amounts of collagen accumulation together with less prominent bridging between portal fields, as indicated by H&E staining (Fig. 4A). In response to BDL, α-SMA immunofluorescence staining indicated that IDO1^−/^^−^ fibrotic mice displayed reduced hepatic levels of pro-fibrogenic factor α-SMA compared to WT fibrotic mice (Fig. 4A, B). Consistently, serum AST and ALT levels were found to be significantly decreased in IDO1^−/−^ fibrotic mice compared to WT fibrotic mice, evidencing that hepatocytes injury were ameliorated in IDO1-deficient mice (Fig. 4C). In line with these results, we proposed that deficiency of IDO1 could protect mice from liver fibrosis induced by BDL.

### Deficiency of IDO1 helped to promote hepatic, splenic DCs maturation in response to liver fibrosis induced by BDL

**Fig. 5:**
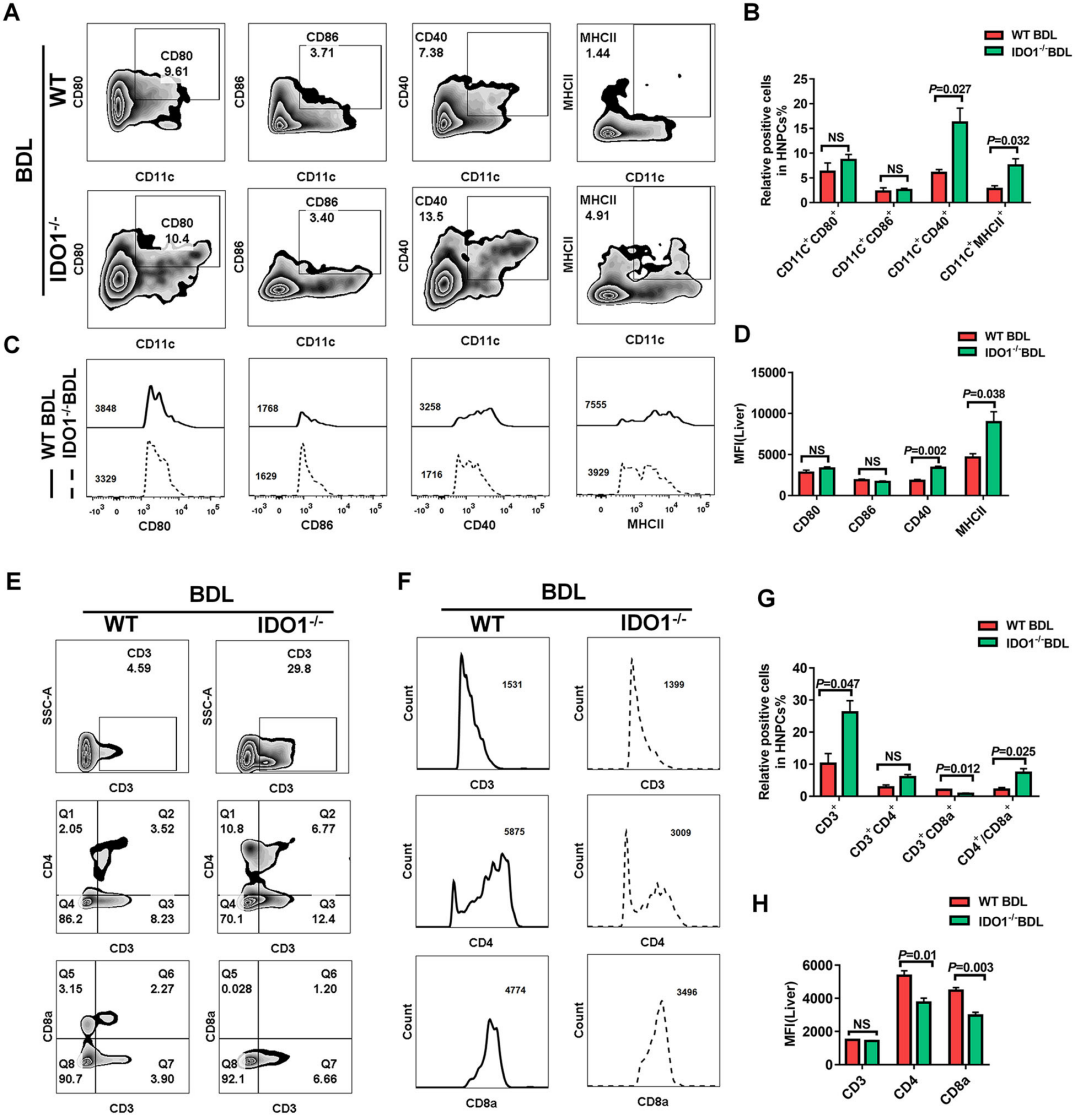
Deficiency of IDO1 helped to promote hepatic, splenic DCs maturation in response to liver fibrosis induced by BDL. **A** Frequencies of hepatic CD11c^+^CD80^+^, CD11c^+^CD86^+^, CD11c^+^MHCII^+^, and CD11c^+^CD40^+^ cells in WT and IDO1^−/^^−^ mice was determined by flow cytometry and summarized data were listed on right (**B**) (*n* ≥ 3). **C** Activation marker expression on hepatic DCs in WT and IDO1^−/−^ mice were measured by flow cytometry and summarized data were listed on right (**D**) (*n* ≥ 3). **E** Frequencies of CD3^+^, CD3^+^CD4^+^, CD3^+^CD8^+^ T cells in hepatic NPCs were assessed by flow cytometry and summarized data were listed on right (**G**) (*n* ≥ 3). **F** MFI of CD3, CD4, CD8a in hepatic NPCs were measured by flow cytometry and summarized data were listed on right (**H**) (*n* ≥ 3). The data are presented as the means ± SEM. *P* < 0.05 is considered statistically significant. Statistical analysis was analyzed by independent-samples *t*-test.

Since no obvious pathological differences in liver tissues were seen between WT and IDO1^−/−^ mice in the absence of BDL challenged. The primary concern of subsequent study was to observe the difference between WT and IDO1^−/−^ mice in response to BDL-induced liver fibrosis. Consequently, we further made a comparison between the maturation status of hepatic CD11c^+^DCs in WT and IDO1^−/−^ mice after induction of liver fibrosis by BDL. Flow cytometry revealed that a significant increase in the frequencies of CD11c^+^MHCII^+^ and CD11c^+^CD40^+^ cells in hepatic NPCs from IDO1^−/−^ fibrotic mice compared to those from WT fibrotic mice, and deficiency of IDO1 enhanced the expression levels of activation markers CD40 and MHCII on hepatic CD11c^+^DCs. Again, the yield of CD11c^+^CD80^+^, CD11c^+^CD86^+^cells did not vary between hepatic NPCs harvested from IDO1^−/−^ fibrotic mice, or WT fibrotic mice and knock out of IDO1 did not influence the expression levels of CD80 and CD86 on hepatic CD11c^+^DCs during liver fibrosis induced by BDL (Fig. 5A–D). Additionally, knock out of IDO1 induced a distinct increase in the frequency of CD3^+^ T cells, and the ratio of CD4^+^/CD8^+^, whereas reduced the frequency of CD8^+^ T cells in hepatic NPCs under the condition of liver fibrosis. Though hepatic NPCs derived from IDO1^−/−^ fibrotic mice expressed similar levels of CD3 as those from WT fibrotic mice, they expressed lower levels of CD4 and CD8a (Fig. 5E–H).

Furthermore, splenic leukocytes in IDO1^−/−^ fibrotic mice had a higher percentage of CD11c^+^MHCII^+^, CD11c^+^CD40^+^, CD11c^+^CD80^+^, and CD11c^+^CD86^+^ cells compared with WT fibrotic mice. Meanwhile, splenic CD11c^+^DCs expressed a higher level of CD40, suggestive of their hyper-activated status (Fig. S5A–C). Moreover, splenic CD11c^+^DCs gained enhanced capacity to activate CD3^+^, CD3^+^CD8^+^ T cells and resulted in a decreased ratio of CD4^+^/CD8^+^ in IDO1^−/−^ fibrotic mice compared to WT fibrotic mice, whereas IDO1^−/−^ fibrotic mice had a lower frequency in splenic and thymic CD3^+^CD4^+^ T cells compared with WT fibrotic mice (Fig. S5A, D, F). Moreover, splenic leukocytes isolated from IDO1^−/−^ fibrotic mice expressed similar levels of CD3, CD4, CD8a as those from WT fibrotic mice (Fig. S5E). No differences in the frequencies of thymic CD3^+^, CD3^+^CD8^+^ T cells were found between IDO1^−/−^ fibrotic mice and WT fibrotic mice (Fig. S5F). The MFI of thymic leukocytes in IDO1^−/−^ fibrotic mice exhibited a lower level of CD8a as compared to WT fibrotic mice, while they presented similar expression levels of CD3, CD4 (Fig. 5G).

Collectively, these results suggest that IDO1 appeared crucial for trafficking of these immune cells during liver fibrosis. Moreover, enhanced maturation markers expression level of DCs, subsequent DCs-mediated T-cell proliferation may partially account for the amelioration of liver fibrosis in IDO1^−/−^ fibrotic mice.

### Overexpression of IDO1 led to worse liver fibrosis induced by BDL in mice

Based on the above experimental results, we postulated that overexpression of IDO1 might lead to exacerbated liver fibrosis. To test this, mice were injected with recombinant adeno‐associated virus serotype 9 vectors carrying IDO1(AAV-IDO1) with a TBG promoter to overexpressing IDO1 in liver tissues specifically, and mice injected with empty recombinant adeno‐associated virus serotype 9 served as negative control (AAV-NC). As presented in Fig. S6C–D, overexpression of IDO1 was confirmed in AAV-IDO1 infected mice livers derived therein. Similarly, in the absence of damage, serum levels of AST, ALT and H&E staining had no obvious differences between AAV9-NC and AAV-IDO1 infected mice (Fig. S6A, B).

**Fig. 6:**
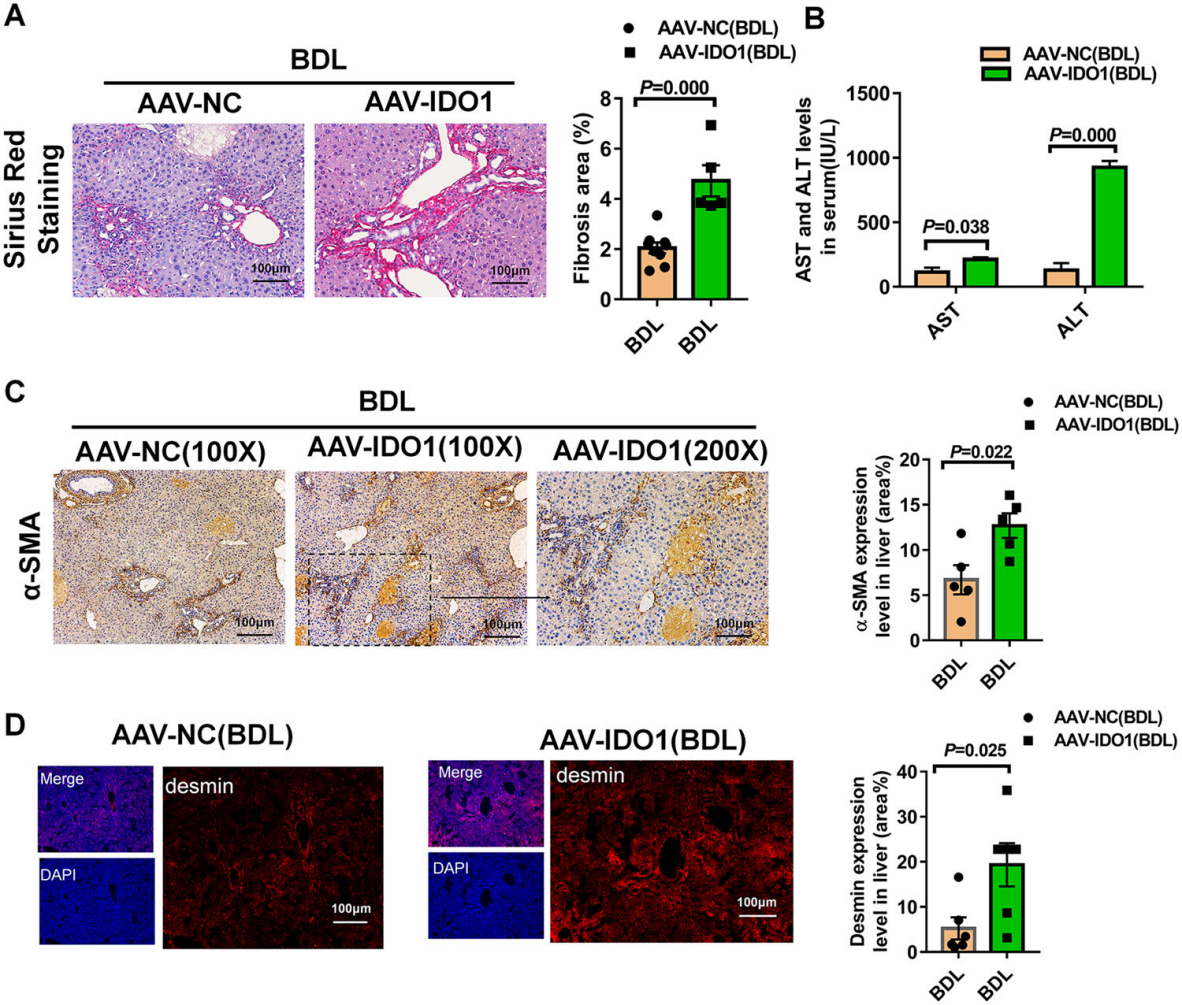
Overexpression of IDO1 led to worse liver fibrosis induced by BDL in mice. **A** Sirius Red staining of liver sections in AAV-NC, AAV-IDO1 infected mice (*n* ≥ 5). **B** Liver injury was assessed by measuring serum ALT and AST levels in AAV-NC, AAV-IDO1 infected mice (*n* ≥ 3). **C** α-SMA immunohistochemistry staining of liver sections in AAV-NC, AAV-IDO1 infected mice. α-SMA quantification of positive-stained areas was shown in the graph (*n* = 5). **D** Desmin immunofluorescence staining of liver sections in AAV-NC, AAV-IDO1 infected mice. Desmin quantification of positive-stained areas was shown in the graph (*n* = 6). In all panels, scale bars, 100 mm. The data are presented as the means ± SEM. *P* < 0.05 is considered statistically significant. Statistical analysis was analyzed by independent-samples *t-*test.

Consistent with our hypothesis, overexpression of IDOI led to worse hepatocellular injury as indicated by stronger increased levels of serum AST, ALT (Fig. 6B). As expected, Sirius Red staining also proved that IDO1 overexpression led to worse liver fibrosis (Fig. 6A). Consistently, liver tissues derived from fibrotic mice challenged with AAV-IDO1 had more α-SMA, desmin-positive cells than those challenged with AAV-NC, respectively (Fig. 6C, D). These results suggested that overexpression of IDO1contributed to the exacerbation of BDL-induced fibrosis in mice.

### Overexpression of IDO1 suppressed the maturation of hepatic, splenic DCs during liver fibrosis induced by BDL

**Fig. 7:**
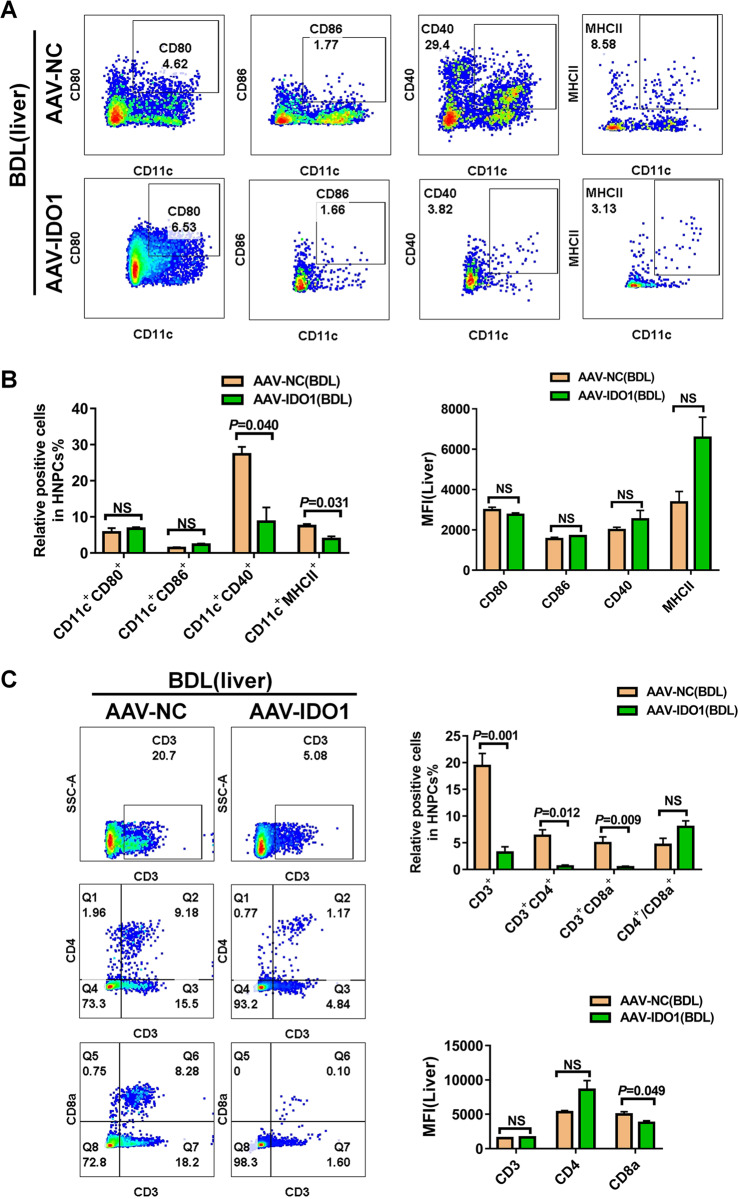
Overexpression of IDO1 suppressed the maturation of hepatic DCs during liver fibrosis induced by BDL. **A** Frequencies of hepatic CD11c^+^CD80^+^, CD11c^+^CD86^+^, CD11c^+^MHCII^+^, CD11c^+^CD40^+^ cells in AAV-NC, AAV-IDO1 infected mice were determined by flow cytometry. **B** Summarized data of **A** were listed below (*n* ≥ 3). **C** Frequencies of hepatic CD3^+^, CD3^+^CD4^+^, CD3^+^CD8^+^ T cells in AAV-NC, AAV-IDO1 infected mice were determined by flow cytometry. Summarized data of **C** were listed on the right (*n* ≥ 3). The data are presented as the means ± SEM. *P* < 0.05 is considered statistically significant. Statistical analysis was analyzed by independent-samples *t-*test.

To gain a deeper insight into the effect of IDO1 expression level on the maturation status of DCs during liver fibrosis, flow cytometry was performed to identify the changes of hepatic, splenic leukocytes in mice injected with AAV-IDO1 or AAV-NC. As shown in Fig. 7A, B, hepatic NPCs in fibrotic mice injected with AAV-IDO1 had lower frequencies of CD11c^+^MHCII^+^, CD11c^+^CD40^+^cells compared with fibrotic mice injected with AAV-NC, whereas overexpression of IDO1 had no effect on the frequencies of CD11c^+^CD80^+^ and CD11c^+^CD86^+^ cells in the livers of fibrotic mice. Moreover, we failed to detect any difference in the expression levels of the activation markers CD80, CD86, CD40, and MHCII on CD11c^+^DCs in fibrotic livers after overexpression IDO1. Hepatic NPCs obtained from fibrotic mice challenged with AAV-IDO1 revealed a robust reduction in the frequencies of CD3^+^, CD3^+^CD4^+^, CD3^+^CD8^+^ T cells compared to those mice challenged with AAV-NC, whereas the ratio of hepatic CD4^+^/CD8^+^ did not show any difference between fibrotic mice challenged with AAV-IDO1 and AAV-NC (Fig. 7C).

Similarly, overexpression of IDO1 led to a strong reduction in the frequencies of splenic CD11c^+^MHCII^+^, CD11c^+^CD40^+^cells and CD3^+^, CD3^+^CD4^+^ T cells as well as the ratio of CD4^+^/CD8^+^in fibrotic mice induced by BDL. However, neither the frequencies of CD11c^+^CD80^+^, CD11c^+^CD86^+^ cells, nor CD3^+^CD8^+^ T cells had a change in the spleen of fibrotic mice under the condition of overexpression IDO1 (Figs. S7A–C and S8A–C). We also failed to detect any difference in the expression levels of the activation markers CD80, CD86, CD40, and MHCII on splenic CD11c^+^DCs in fibrotic mice after overexpression IDO1 (Fig. S7C). Of note, we observed a significant increase in the frequencies of CD3^+^, CD3^+^CD4^+^, CD3^+^CD8^+^ T cells in thymic leukocytes from fibrotic mice challenged with AAV-IDO1 compared to those challenged with AAV-NC (Fig. S9A–D). These findings, together with our earlier results, suggested that the decreased maturation of hepatic, splenic DCs and subsequent reduced proliferation rate of T cells might account for the exacerbated liver fibrosis in IDO1-overexpression mice.

## Discussion

With changes of social environmental factors, diet structure including obesity, viral infection and alcohol consumption, the incidence rate of liver fibrosis had grown each year^5^. Hepatic fibrosis is irreversible without any specific clinical symptoms, the risk of cirrhosis could be lessened through prevention and early intervention for liver fibrosis^5^. However, there are presently no effective drugs available to cure liver fibrosis. To explore specific clinical treatments to prevent or reverse liver fibrosis, it is intense to better understand the underlying mechanisms of liver fibrosis.

Previously, we observed that serum IDO1 level was decreased in patients with HBV-induced cirrhosis as compared to healthy volunteers, whereas serum IDO1 was dramatically increased in fibrotic mice induced by CCL4^14^. In a recent study by our group, we reported that IDO1 acted as an important factor that triggered RNS stress, contributed to ferritin degradation and further caused ferroptosis in ConA-induced liver injury^15^. However, it was reported that IDO might have a protective effect against hepatic fibrosis in HFD-induced liver injury model^27^. As we all know, there are many methods to establish liver fibrosis model in experimental study, and the pathogenesis of each animal model is different. CCL4 is metabolized in the liver by cytochrome P450 enzymes and converted to a highly reactive tri-chloromethyl (CCL3^●^) radical, ultimately leading to hepatotoxic damage, inflammation and fibrosis and most intraperitoneal mice models described only reach a stage of CLD that could be defined as fibrosis or early cirrhosis^28^. Common bile duct ligation (BDL) is a model of secondary biliary cirrhosis that can be performed both in rats and mice. The surgical obstruction of the common bile duct causes bile to accumulate in the liver, leading to hepatic injury, inflammation and, ultimately, fibrosis and cirrhosis^29,30^. HFD-fed animal models were mainly used for the characterization of potential drug effects on body weight, hepatic steatosis, and, to some extent, inflammation^31^. Given these points, it is not difficult to understand why different animal models have different experimental results. Additionally, published literatures showing that the inhibition of IDO1 may be used to treat chronic HCV patients in vivo and the inhibition of IDO1 could alleviate murine liver damage with the reduction of inducible nitric oxide synthase and 3-nitrotyrosine expression^15,32^. It has been reported that IDO1 functions as a pro-fibrogenesis factor in CCL4-induced liver fibrosis^33^^,34^. Further support to this notion comes from present work, the dramatically upregulated expression of hepatic IDO1 after BDL was identified in WT mice, and its role during the development of liver fibrosis was repeated validations by experiments applying IDO1-deficient and IDO1-overexpression mice. Our results indicated IDO1-deficient fibrotic mice exhibited milder liver fibrosis than WT fibrotic mice, whereas overexpression of IDO1 led to worsening liver fibrosis. Thus, our findings suggested that IDO1 may promote BDL-induced liver fibrosis.

Increasing pieces of evidences indicated that the pathogenesis of liver fibrosis related to the changes in immune function^9,10,35^. It is no doubt that the immunologic reactions response to hepatic fibrosis are complicated and incompletely clarified. Recruitment of immune cells, like DCs or T cells, is a crucial process for the initiation of inflammation, wound healing and hepatic fibrosis, as well^36^. As the principal antigen-presenting cells, DCs play a central role in adaptive and innate immunity, including capturing, processing and presenting antigenic material to T lymphocytes^37,38^. Meanwhile, it was reported that IDO1 was critically involved in inflammatory cells infiltration, including macrophages, DCs, T cells, etc.^39–43^.

Since both IDO1 and DCs play a crucial function in liver inflammation and immunity, we postulated IDO1 might play a critical role in modulating DCs in BDL-mediated liver fibrosis. Indeed, apart from the elevated expression of IDO1 in fibrotic livers, the frequency of hepatic CD11c^+^DCs reduced from ~39.1% in controls to ~16.2% in BDLs and undergo a transformation in mature phenotype in BDL-induced liver fibrosis. In fibrotic livers, the percentage of CD11c^+^MHCII^+^, CD11c^+^CD40^+^ cells were significantly decreased as compared to normal livers, accompanied by a lower MFI of co-stimulatory molecules (MHCII, CD40), which indicated that hepatic CD11c^+^DCs exhibited tolerogenic properties in mice treated with BDL. The immature DCs are characterized by low expression of co-stimulatory molecules (MHCII, CD40) accompanied by low T-cell activation potential^44^. Consistently, hepatic CD3^+^, CD3^+^CD4^+^ T cells were reduced remarkedly in fibrotic livers. This hypothesis was further validated in our research reported here, in IDO1-KO mice model, where exhibited markedly attenuated liver fibrosis, together with an elevated percentage of CD11c^+^MHCII^+^, CD11c^+^CD40^+^ cells in hepatic NPCs of fibrotic livers. By contrast, overexpression of IDO1 significantly aggravated liver fibrosis, accompanied by a dramatically reduced percentage of CD11c^+^MHCII^+^, CD11c^+^CD40^+^ cells and CD3^+^, CD3^+^CD4^+^ T cells in hepatic NPCs of fibrotic livers. Above results corroborate our previous hypothesis suggest that the observed changes in immune cells recruitment of DCs and T-cell presented a transform in hepatic inflammatory microenvironment might cause by the different expression level of IDO1 during BDL-induced liver fibrosis.

Additionally, published literatures indicated that the development of liver fibrosis might lead to pathological changes in spleen^45–47^. It was well-established that spleen was mainly composed of lymphoid tissue, which served as the center of the blood defense system, and thus was essential for eliminating pathogens and immune homeostatic^48–50^. Besides facilitated the recruitment of immune cells, spleen tyrosine kinase signaling pathway promoted the activation of HSCs and exacerbated liver fibrosis^51^. Consequently, flow cytometry was also performed on splenic leukocytes to address whether splenic immune response contributes to the pro-fibrogenic role of IDO1 during BDL-induced liver fibrosis. Surprisingly, in WT mice model, splenic leukocytes exhibited a significant decrease in the frequency of CD11c^+^ DC cells and CD11c^+^CD80^+^, CD11c^+^CD86^+^, CD11c^+^MHCII^+^, CD11c^+^CD40^+^ cells, whereas we failed to detect any changes of splenic CD3^+^, CD3^+^CD4^+^ T cells infiltration upon BDL-operated in our study. Furthermore, fibrotic mice depleted of IDO1 had significantly expanded numbers of CD11c^+^CD80^+^, CD11c^+^CD86^+^, CD11c^+^MHCII^+^, CD11c^+^CD40^+^ cells and increased CD3^+^, CD3^+^CD8^+^ T cells when compared to WT model mice, suggestive that splenic DCs gained enhanced capacity to stimulate CD3^+^, CD3^+^CD8^+^ T cells. Moreover, overexpression of liver‐specific IDO1 diminished numbers of splenic CD11c^+^MHCII^+^, CD11c^+^CD40^+^ cells and reduced CD3^+^, CD3^+^CD4^+^ T cells infiltration subsequently during BDL-induced hepatic fibrosis. From these statistical analyses, we demonstrated that pathophysiological changes in liver fibrosis might result in spleen immune environment variance, including the limited phenotypic activation of DCs and subsequent T cells. Thus, our studies provided a valuable new insight that combining analysis of liver and spleen might present higher diagnostic and therapeutic performance than liver alone in patients with liver cirrhosis.

In conclusion, IDO1 was elevated significantly in fibrotic livers after BDL-operated and involved in the progression of immune cells recruitment and fibrosis; IDO1 may be a potential target for the treatment of liver fibrosis. Moreover, amelioration of immune response in the hepatic, splenic microenvironment by targeting IDO1 might be essential for the treatment effects on liver fibrosis.

## Materials and methods

### Animals

We certified that the use of all mice involved in this project, including the number of animals, species used, or procedures performed were carried out according to the provisions of the Animal Welfare Act, PHS Animal Welfare Policy, the principles of the “NIH Guide for the Care and Use of Laboratory Animals,” and approved by the National Institutional Animal Care and Ethical Committee of Guangzhou University of Chinese Medicine (S2017040, S2017043). Male C57/BL6 mice, aged 6–7 weeks were purchased from Jinan Peng Yue Experimental Animal Breeding Co. LTD and bred in pathogen-free facilities at Southern Medical University. Male IDO1^−/−^ mice (B6·129-IDO1^tm1Alm^/J, Jackson Laboratory, 005867) were genotyped according to the Jackson Laboratory (Bar Harbor, ME, USA) technical support.

### BDL model

Mice were randomly assigned. The common bile duct was doubly ligated with 6–0 absorbable sutures and transected between the ligated sites in model mice. Sham-operated mice were performed the same surgical procedures without BDL. Mice were sacrificed after 3 weeks, and livers, serum samples were collected, processed for further analysis.

### In vivo AAV9‐mediated liver‐specific IDO1 overexpression

In vivo IDO1 overexpression was achieved via AAV9 vectors. Recombinant adeno‐associated virus serotype 9 vectors carrying IDO1 (AAV-IDO1) with a TBG promoter were purchased from GeneChem Co., Ltd (Shanghai, China). The custom-made adenoviral vector without carrying IDO1 served as negative control (AAV-NC). These adenoviral vectors were injected into mice (3 × 10^11^ vector genomes/mouse) to overexpress IDO1 via tail vein. Twenty-eight days after AAV9 delivery, overexpression of IDO1 was confirmed in livers tissues by immunofluorescence staining and western blot analysis. The remaining mice were subjected to BDL challenge.

### Flow cytometry

Six- and four-color flow cytometry were performed on BD LSRFortessa X-20. We adjusted voltages based on unstained cells, and compensation was performed according to single-stained-positive control for each color. Dead cells were excluded by 7-aminoactinomycin D (BD Biosciences) and added 0.5 µl CD16/32 (BD Biosciences) to block nonspecific Fc-mediated interactions.

Six-color staining was performed by using the following panel of mAbs to quantitate DCs immunophenotyped: PE-Cy7 conjugated anti-CD11c (BD Biosciences, San Diego, CA, USA), BV421 conjugated anti-CD80 (BD Biosciences, San Diego, CA, USA), BV605 conjugated anti-CD86 (BD Biosciences, San Diego, CA, USA), PE conjugated anti-CD40(BD Biosciences, San Diego, CA, USA), Alexa Flour488 anti-MHCII(BD Biosciences, San Diego, CA, USA).

Four-color staining was performed to address the proliferation levels of T lymphocytes by using the following panel of mAbs: APC-Cy7 conjugated anti-CD3 (BD Biosciences, San Diego, CA, USA), PE conjugated anti-CD4 (BD Biosciences, San Diego, CA, USA), FITC conjugated anti-CD8a (BD Biosciences, San Diego, CA).

### Biochemical analyses

Blood samples were collected through cardiac puncture for biochemical analysis before perfusion. After placing at room temperature for 4 h, centrifugation at 3000 × *g* for 15 min, the supernatant serum was harvested and stored at −80 °C until the measurement. The serum ALT and AST concentration were assessed by AST/GOT Assay Kit and ALT/GPT Assay Kit (Nanjing Jiancheng Bioengineering Institute, China) following the manufacturer’s directions.

### Western blot analysis

Liver tissues were extracted and dissolved in 1× RIPA lysis buffer containing a protease inhibitor cocktail (Sigma, USA) and phosphatase inhibitor cocktail (Sigma, USA). BCA assay Protein Quantitation Kit was used to detected protein lysate concentrations. The following primary antibodies were used for western blotting analysis: IDO1 (rabbit, 1:1000, Cell Signaling Technology), α-SMA (rabbit, 1:1000, Cell Signaling Technology), CD11c (rabbit, 1:1000, Cell Signaling Technology), GAPDH (rabbit, 1:1000, Proteintech), β-actin (mouse, 1:2000, Affinity Biosciences).

### Histology and immunofluorescence, immunohistochemistry staining

For histology, liver samples were fixed with 4% paraformaldehyde, conventional dehydrated, embedded in paraffin sections, cut into 4 μm-thick sections. H&E and Sirius red staining were performed according to standard directions.

For immunofluorescence staining, liver tissues were immersed in 4% paraformaldehyde for 48 h. Followed by 15%, 30% sucrose gradient dehydrated before the liver samples sink to the bottom completely. Frozen OCT-embedded tissues were sliced into 14 μm-thick sections. Blocking the liver sections with a blocking buffer containing 0.01 M PBS, 0.1% Triton X-100 and 5% goat serum solution for 60 min at room temperature. Then the slices were incubated with appropriate primary antibodies at 4 °C overnight, followed stained with Alexa488/594 conjugated secondary antibodies (1:300, Invitrogen) for 1 h at room temperature. Finally, sections were counterstained with 4, 6-diamidino-2-phenylindole (DAPI, Solarbio Life Science, China). The following primary antibodies were used for immunofluorescence: desmin (rabbit, 1:200, Cell Signaling Technology), IDO1 (mouse, 1:50, Santa Cruz Biotechnology), α-SMA (rabbit, 1:100, Proteintech), goat anti-mouse/rabbit Alexa Fluor 488-conjugated IgG (1:300, Invitrogen) or goat anti-rabbit/mouse Alexa Fluor 568-conjugated IgG (1:300, Invitrogen).

For immunohistochemistry, conventional dewaxing, hydration and antigen retrieval according to directions by steps on the immunohistochemistry kits. Incubated with 0.3% H_2_O_2_ for 10 min at room temperature to block endogenous peroxidase and then incubated with blocking buffer to lessen nonspecific binding sites for 60 min at room temperature. Paraffin‐embedded slices were then stained with the appropriate primary antibodies overnight at 4 °C, followed by staining with HRP anti-rabbit IgG or anti-mouse IgG antibodies for 1 h at room temperature. Next steps were performed following the instructions of GTvision immunohistochemical Kit (Gene Tech, Shanghai, China). The following primary antibodies were used for immunohistochemistry: CD11c (rabbit,1:1000, Cell Signaling Technology), α-SMA (rabbit, 1:1000, Cell Signaling Technology).

### Statistical analyses

All values were presented as means and SEM. Statistical analyses were performed through SPSS software. Data were compared using independent-samples *t*-test for two groups or one-way ANOVA for multi-group variable comparisons. In all cases, data from at least three independent experiments were used. A value of *P* < 0.05 is considered statistically significant.

## Supplementary information

Supplementary Figure Legends

S1: Splenic DCs exhibited an immature phenotype in BDL-induced liver fibrosis.

S2: Proliferation rate of splenic T cells after BDL-operated were assessed by flow cytometry.

S3: Proliferation rate of thymic T cells after BDL-operated were assessed by flow cytometry.

S4: Gene identification result of IDO1-/–mice.

S5: Knock out of IDO1 helped to promote splenic DCs maturation and subsequent splenic, thymic T cells in response to liver fibrosis induced by BDL.

S6: The degree of liver injury in AAV9-NC, AAV9-IDO1 infected mice in the absence of damage.

S7: Overexpression of IDO1 suppressed the maturation of splenic DCs during liver fibrosis induced by BDL.

S8: Overexpression of IDO1 suppressed the proliferation rate of splenic T cells during liver fibrosis induced by BDL.

S9: Overexpression of IDO1 suppressed the proliferation rate of thymic T cells during liver fibrosis induced by BDL.
