# Supplementary material for: Indoleamine 2,3-dioxygenase 1 limits hepatic inflammatory cells recruitment and promotes bile duct ligation-induced liver fibrosis
Source: Cell Death Dis. 2021 Jan 7;12(1):16. doi: 10.1038/s41419-020-03277-0 (PMC7791029; doi:10.1038/s41419-020-03277-0)
Supplement: Supplementary file 1 — Supplementary Figure Legends [file 41419_2020_3277_MOESM1_ESM.doc]

**Indoleamine 2, 3-dioxygenase 1 limits hepatic inflammatory cells recruitment and promotes bile duct ligation induced liver fibrosis**

**Chan Mo^a#^, Shuwen Xie ^a#^, Weichao Zhong ^d^, Ting Zeng ^a^, Sha Huang ^a^, Yuqi Lai ^a^, Guanghui Deng ^a^, Chuying Zhou ^a^, Weixin Yan ^a^, Yuyao Chen ^a^, Shaohui Huang ^a^, Lei Gao ^a, b, c *^,**

**Zhiping Lv ^a, **^**

*^a^School of Traditional Chinese Medicine, Southern Medical University, Guangzhou, Guangdong, 510515, PR China*

*^b^ The Key Laboratory of Molecular Biology, State Administration of Traditional Chinese Medicine, School of Traditional Chinese Medicine, Southern Medical University, Guangzhou, Guangdong, 510515, PR China*

*^c^Guangdong Provincial Key Laboratory of Shock and Microcirculation, Southern Medical University, Guangzhou,510515, PR China*

*^d^ Shenzhen Traditional Chinese Medicine Hospital,* *No.1, Fuhua Road, Futian District, Shenzhen, Guangdong, 518033, PR China*

^#^These authors contributed equally to this work.

* Corresponding author

Lei Gao, School of Traditional Chinese Medicine, Southern Medical University;

The Key Laboratory of Molecular Biology, State Administration of Traditional Chinese Medicine, School of Traditional Chinese Medicine, Southern Medical University;

Guangdong Provincial Key Laboratory of Shock and Microcirculation, Southern Medical University; Guangzhou, Guangdong,510515, PR China. Tel: 020 61648641; Fax: 020 61648244,

E-mail address: raygaolei@smu.edu.cn

* *Corresponding author

Zhiping Lv, School of Traditional Chinese Medicine, Southern Medical University,

Guangzhou, Guangdong, 510515, PR China.Tel.:020 61648241; Fax: 020 61648244,

E-mail address: lzp48241@126.com

***Supplementary Figure Legends***

**Figure S1: Splenic DCs exhibited an immature phenotype in BDL-induced liver fibrosis.** (A) Activation marker expression on splenic DCs were measured by flow cytometry. Representative FACS plots showing CD11c^+^CD80^+^, CD11c^+^CD86^+^, CD11c^+^MHCII^+^ and CD11c^+^CD40^+^ cells in WT control and WT model mice and summarized data were listed below (B) (n≥3). (C) MFI of CD80, CD86, CD40 and MHCII in splenic leukocytes were measured by flow cytometry (n≥3). The data are presented as the means ± SEM. *P*<0.05 is considered statistically significant. Statistical analysis was analyzed by independent-samples t test.

**Figure S2: Proliferation rate of splenic T cells after BDL-operated were assessed by flow cytometry.** (A) Frequencies of CD3^+^, CD3^+^CD4^+^, CD3^+^CD8^+^ T cells in splenic leucocytes were assessed by flow cytometry and summarized data were listed below (B, C) (n≥3). (D) MFI were indicated for each histogram and summarized data were listed below (E) (n≥3). *P*<0.05 is considered statistically significant. Statistical analysis was analyzed by independent-samples t test.

**Figure S3: Proliferation rate of thymic T cells after BDL-operated were assessed by flow cytometry.** (A) Frequencies of CD3^+^, CD3^+^CD4^+^, CD3^+^CD8^+^ T cells in thymic leucocytes were assessed by flow cytometry and summarized data were listed below (B, C) (n≥3). (D) MFI were indicated for each histogram and summarized data were listed below (E) (n≥3). *P*<0.05 is considered statistically significant. Statistical analysis was analyzed by independent-samples t test.

**Figure S4:** Gene identification result of IDO1^-/--^mice were validated according to the standard PCR protocols posted on Jackson Laboratory 's official website.

**Figure S5: Knock out of IDO1 helped to promote splenic DCs maturation and subsequent splenic, thymic T cells in response to liver fibrosis induced by BDL.** (A) Frequencies of splenic CD11c^+^CD80^+^, CD11c^+^CD86^+^, CD11c^+^MHCII^+^, CD11c^+^CD40^+^ cells (right) and CD3^+^, CD3^+^CD4^+^, CD3^+^CD8^+^ T cells (left) in WT and IDO1^–/–^mice were determined by flow cytometry and summarized data were listed below (B, D) (n≥3). (C) Activation marker expression on splenic DCs in WT and IDO1^–/–^mice were measured by flow cytometry (n≥3). (E) MFI of CD3, CD4, CD8a in splenic leucocytes were measured by flow cytometry (n≥3). (F) Frequencies of thymic CD3^+^, CD3^+^CD4^+^, CD3^+^CD8^+^ T cells in WT and IDO1^–/–^mice were determined by flow cytometry (n≥3). (G) MFI of CD3, CD4, CD8a in thymic leucocytes were measured by flow cytometry (n≥3). The data are presented as the means ± SEM. *P*<0.05 is considered statistically significant. Statistical analysis was analyzed by independent-samples t test.

**Figure S6: The degree of liver injury in AAV9-NC, AAV9-IDO1 infected mice in the absence of damage.** (A) H&E staining in liver tissues of AAV9-NC, AAV9-IDO1 infected mice in the absence of damage. (B) AST and ALT serum levels in AAV9-NC, AAV9-IDO1 infected mice in the absence of damage(n=3). (C, D) The hepatic IDO1 expression in AAV9-NC, AAV9-IDO1 infected mice in the absence of damage were detected by immunofluorescence staining and immunohistochemistry staining. The data are presented as the means ± SEM. *P*<0.05 is considered statistically significant. Statistical analysis was analyzed by independent-samples t test.

**Figure S7: Overexpression of IDO1 suppressed the maturation of splenic DCs during liver fibrosis induced by BDL.** (A) Frequencies of splenic CD11c^+^CD80^+^, CD11c^+^CD86^+^, CD11c^+^MHCII^+^, CD11c^+^CD40^+^ cells in AAV-NC, AAV-IDO1 infected mice were determined by flow cytometry. (B) Summarized data of (A) were listed below(n≥3). (C) Activation marker expression on splenic DCs in AAV-NC, AAV-IDO1 infected mice were measured by flow cytometry (n≥3). The data are presented as the means ± SEM. *P*<0.05 is considered statistically significant. Statistical analysis was analyzed by independent-samples t test.

**Figure S8:** **Overexpression of IDO1 suppressed the proliferation rate of splenic T cells during liver fibrosis induced by BDL.** (A) Frequencies of splenic CD3^+^, CD3^+^CD4^+^, CD3^+^CD8^+^ T cells (right) and MFI of CD3, CD4, CD8a (left) in splenic leukocytes in AAV-NC, AAV-IDO1 infected mice were measured by flow cytometry. (B-D) Summarized data of (A) were listed below(n≥3). The data are presented as the means ± SEM. *P*<0.05 is considered statistically significant. Statistical analysis was analyzed by independent-samples t test.

**Figure S9: Overexpression of IDO1 suppressed the proliferation rate of thymic T cells during liver fibrosis induced by BDL.** (A) Frequencies of thymic CD3^+^, CD3^+^CD4^+^, CD3^+^CD8^+^ T cells (right) and MFI of CD3, CD4, CD8a (left) in thymic leukocytes in AAV-NC, AAV-IDO1 infected mice were measured by flow cytometry. (B-D) Summarized data of (A) were listed below(n≥3). The data are presented as the means ± SEM. *P*<0.05 is considered statistically significant. Statistical analysis was analyzed by independent-samples t test.
