# Supplementary figures and images for: Indoleamine 2,3-dioxygenase 1 limits hepatic inflammatory cells recruitment and promotes bile duct ligation-induced liver fibrosis
Source: Cell Death Dis. 2021 Jan 7;12(1):16. doi: 10.1038/s41419-020-03277-0 (PMC7791029; doi:10.1038/s41419-020-03277-0)

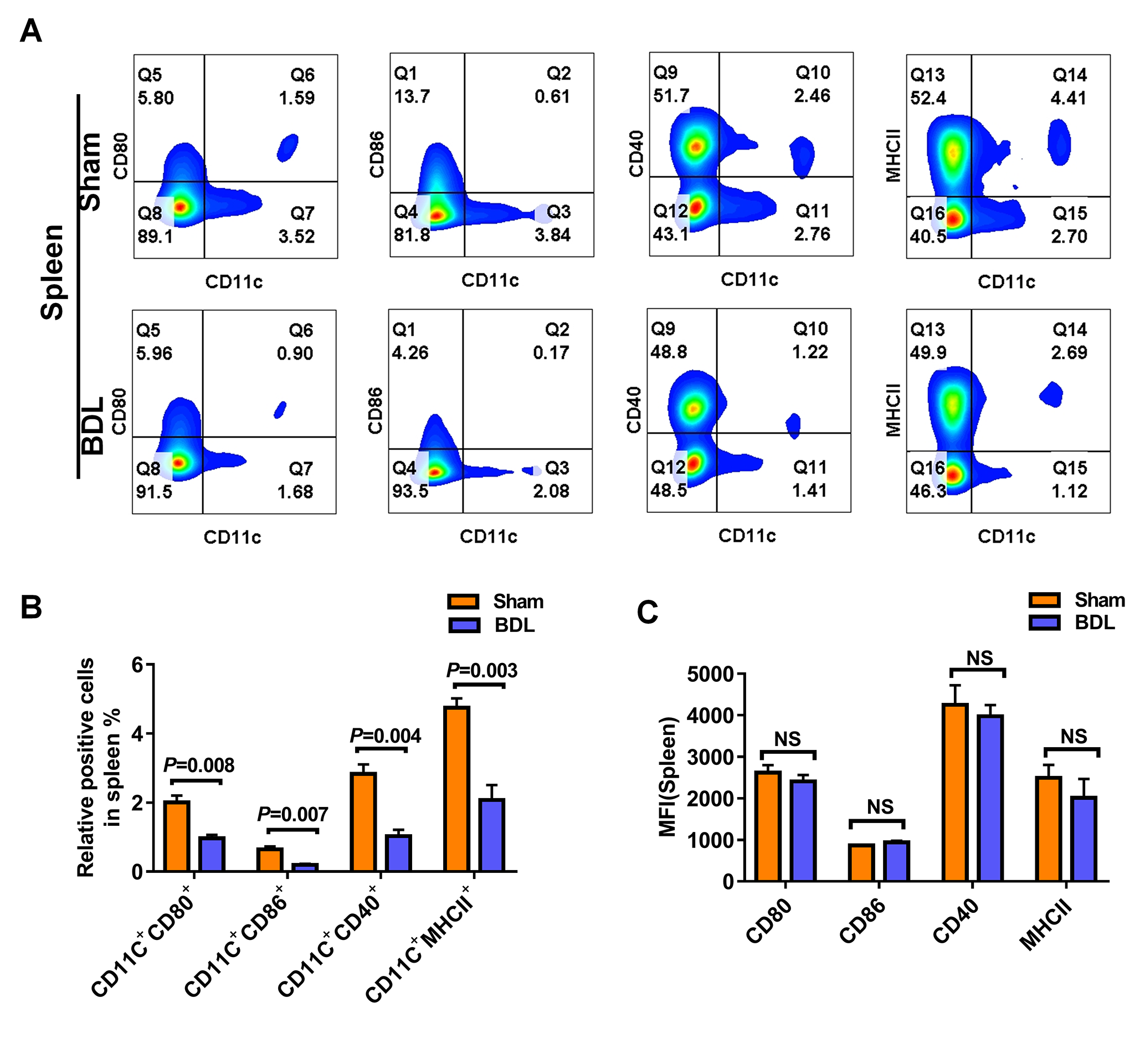

Supplement: Supplementary file 2 — S1: Splenic DCs exhibited an immature phenotype in BDL-induced liver fibrosis. [file 41419_2020_3277_MOESM2_ESM.png]

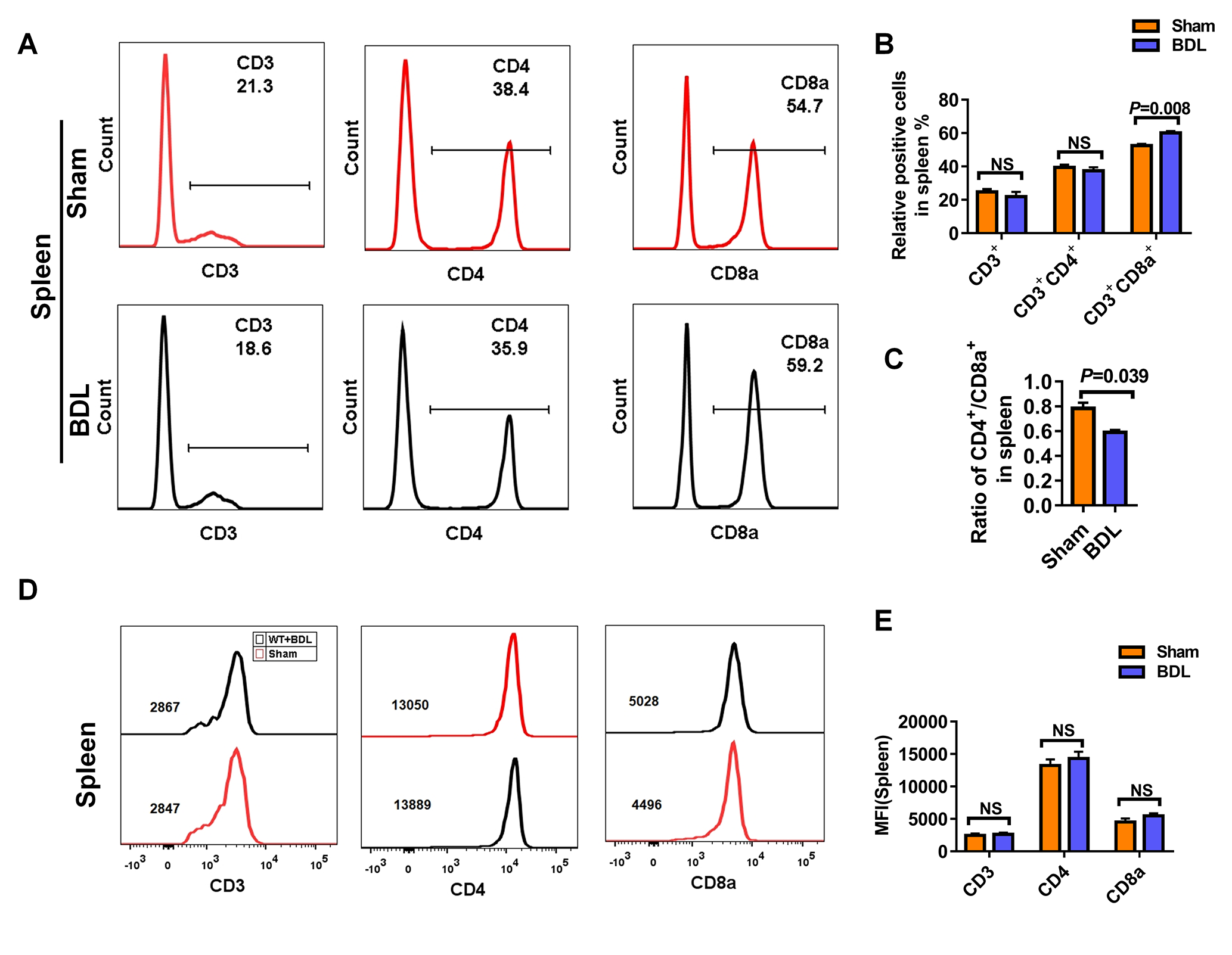

Supplement: Supplementary file 3 — S2: Proliferation rate of splenic T cells after BDL-operated were assessed by flow cytometry. [file 41419_2020_3277_MOESM3_ESM.png]

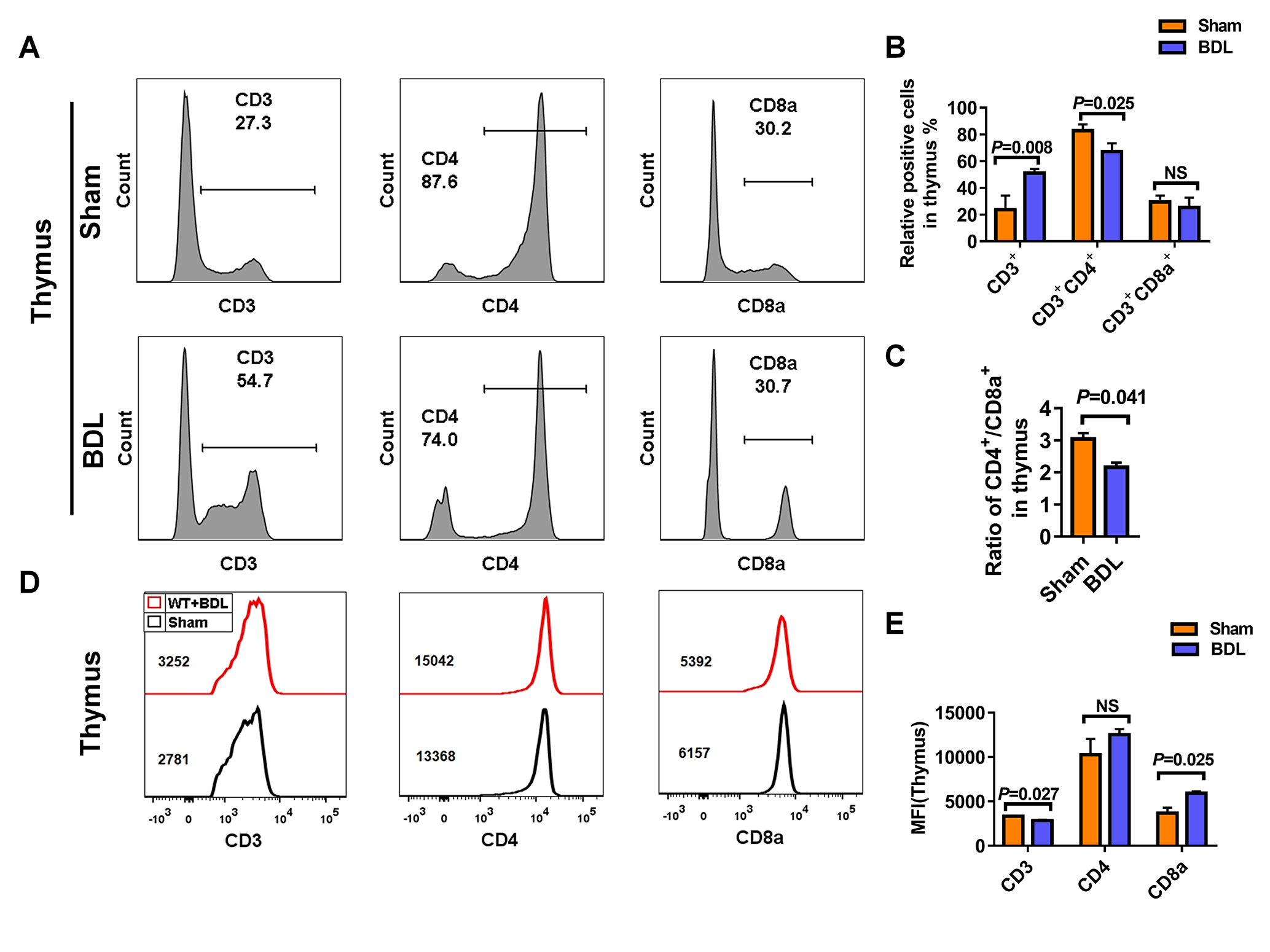

Supplement: Supplementary file 4 — S3: Proliferation rate of thymic T cells after BDL-operated were assessed by flow cytometry. [file 41419_2020_3277_MOESM4_ESM.png]

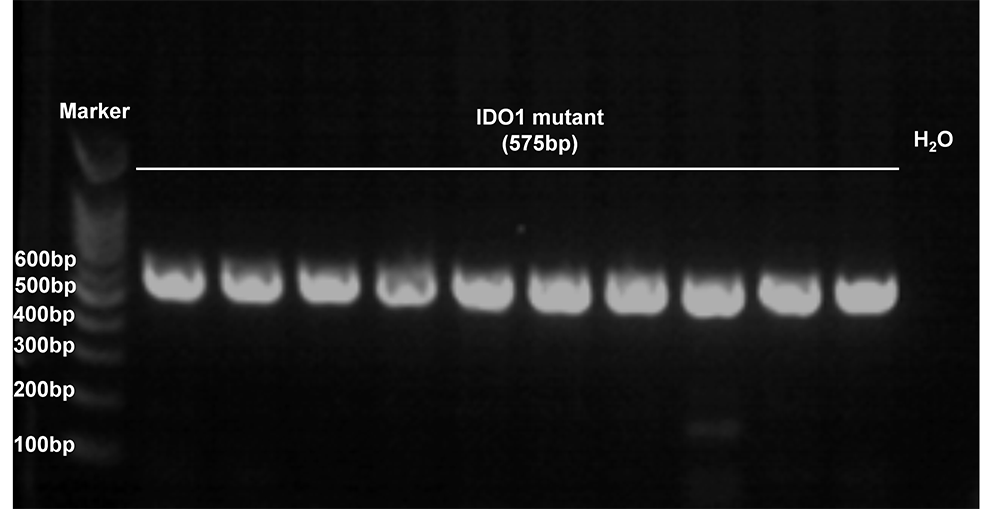

Supplement: Supplementary file 5 — S4: Gene identification result of IDO1-/–mice. [file 41419_2020_3277_MOESM5_ESM.png]

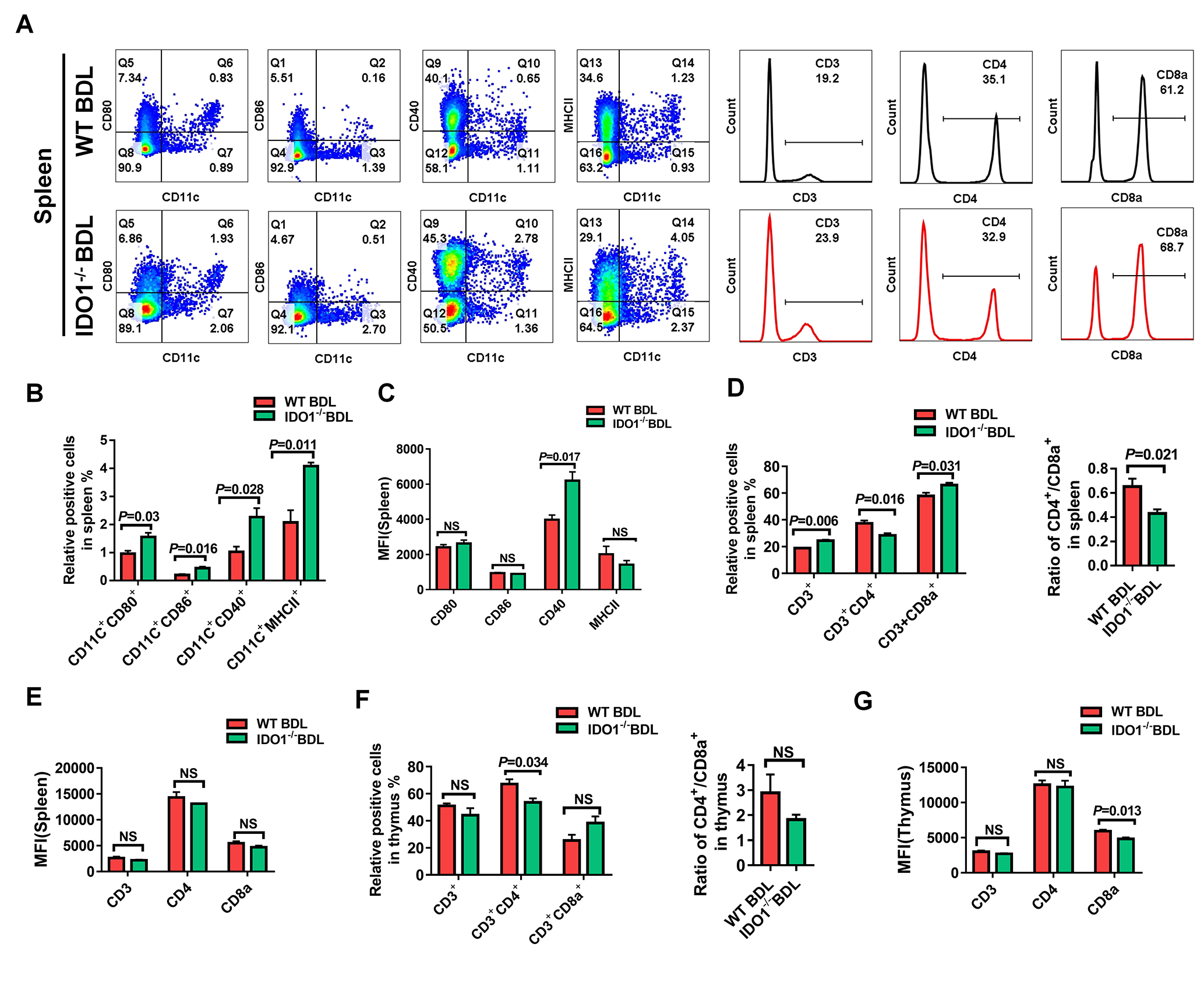

Supplement: Supplementary file 6 — S5: Knock out of IDO1 helped to promote splenic DCs maturation and subsequent splenic, thymic T cells in response to liver fibrosis induced by BDL. [file 41419_2020_3277_MOESM6_ESM.png]

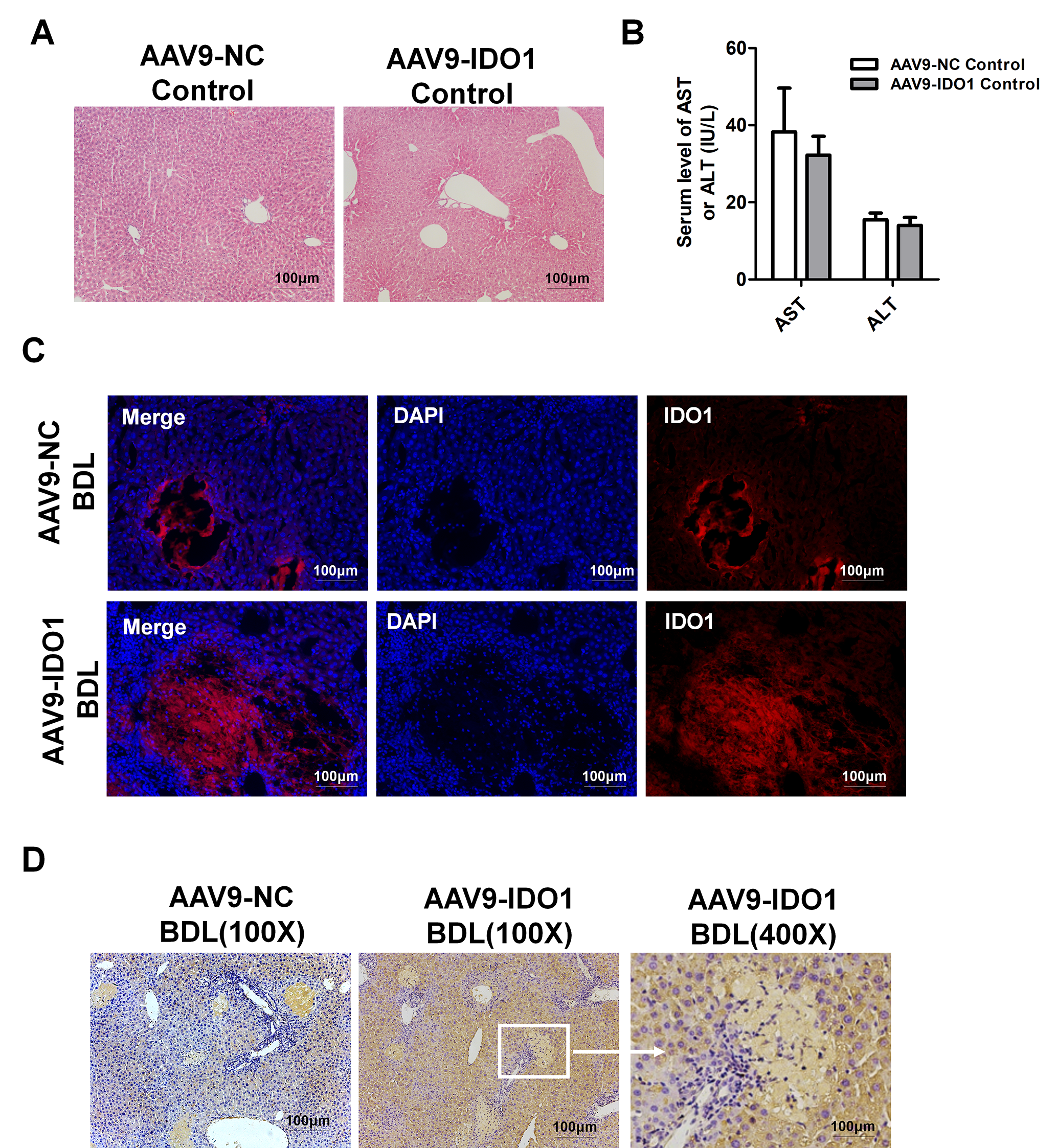

Supplement: Supplementary file 7 — S6: The degree of liver injury in AAV9-NC, AAV9-IDO1 infected mice in the absence of damage. [file 41419_2020_3277_MOESM7_ESM.png]

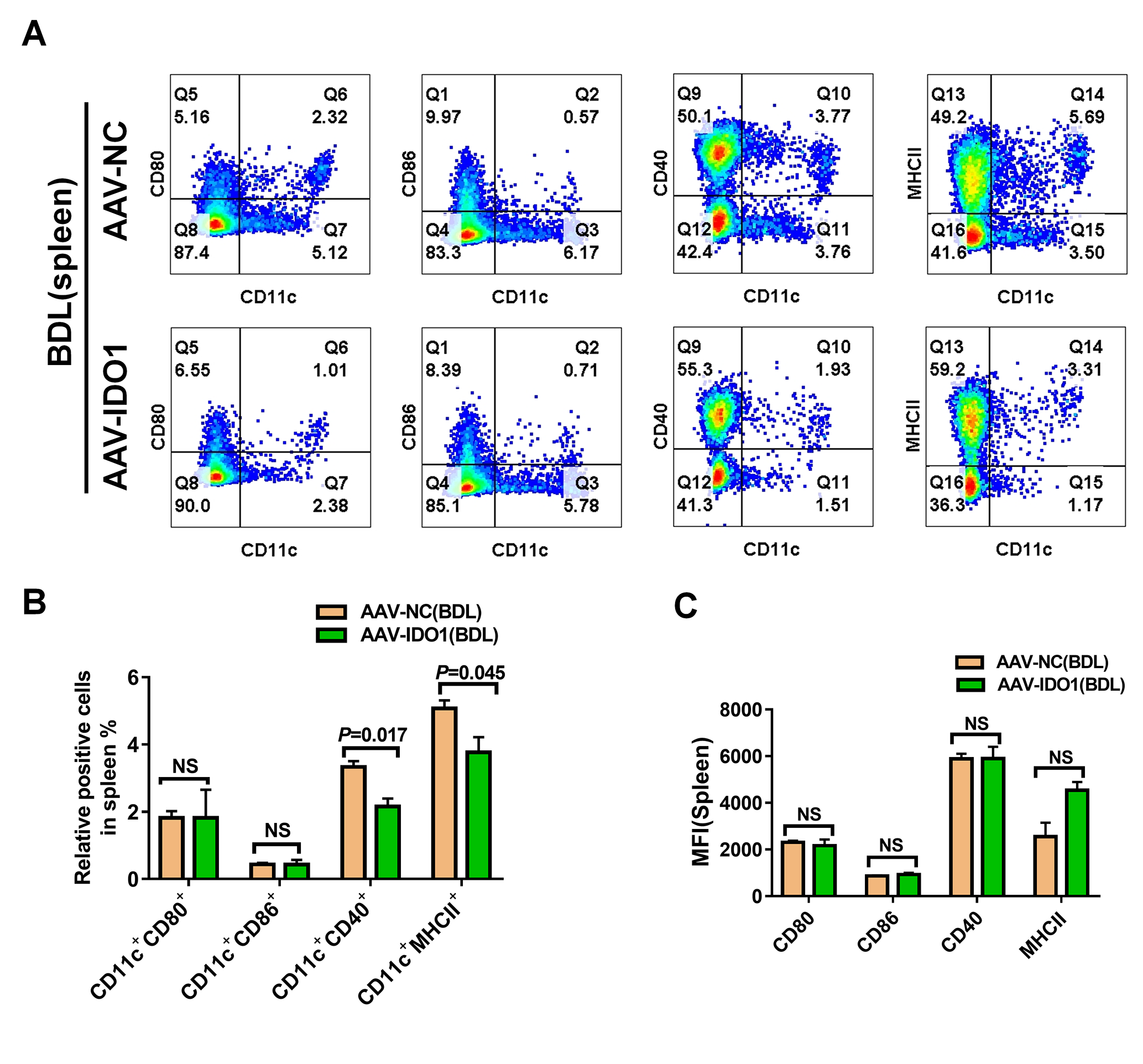

Supplement: Supplementary file 8 — S7: Overexpression of IDO1 suppressed the maturation of splenic DCs during liver fibrosis induced by BDL. [file 41419_2020_3277_MOESM8_ESM.png]

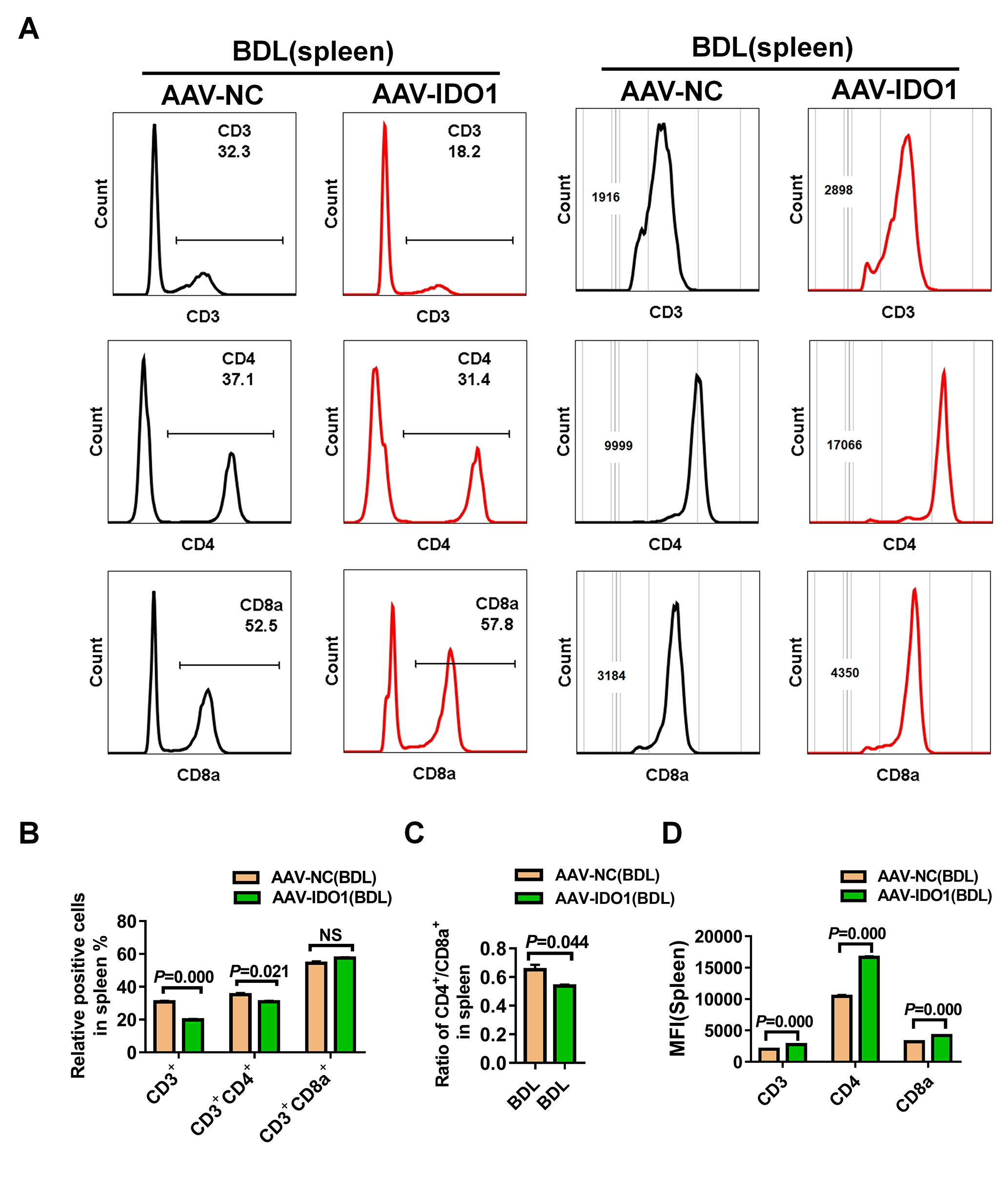

Supplement: Supplementary file 9 — S8: Overexpression of IDO1 suppressed the proliferation rate of splenic T cells during liver fibrosis induced by BDL. [file 41419_2020_3277_MOESM9_ESM.png]

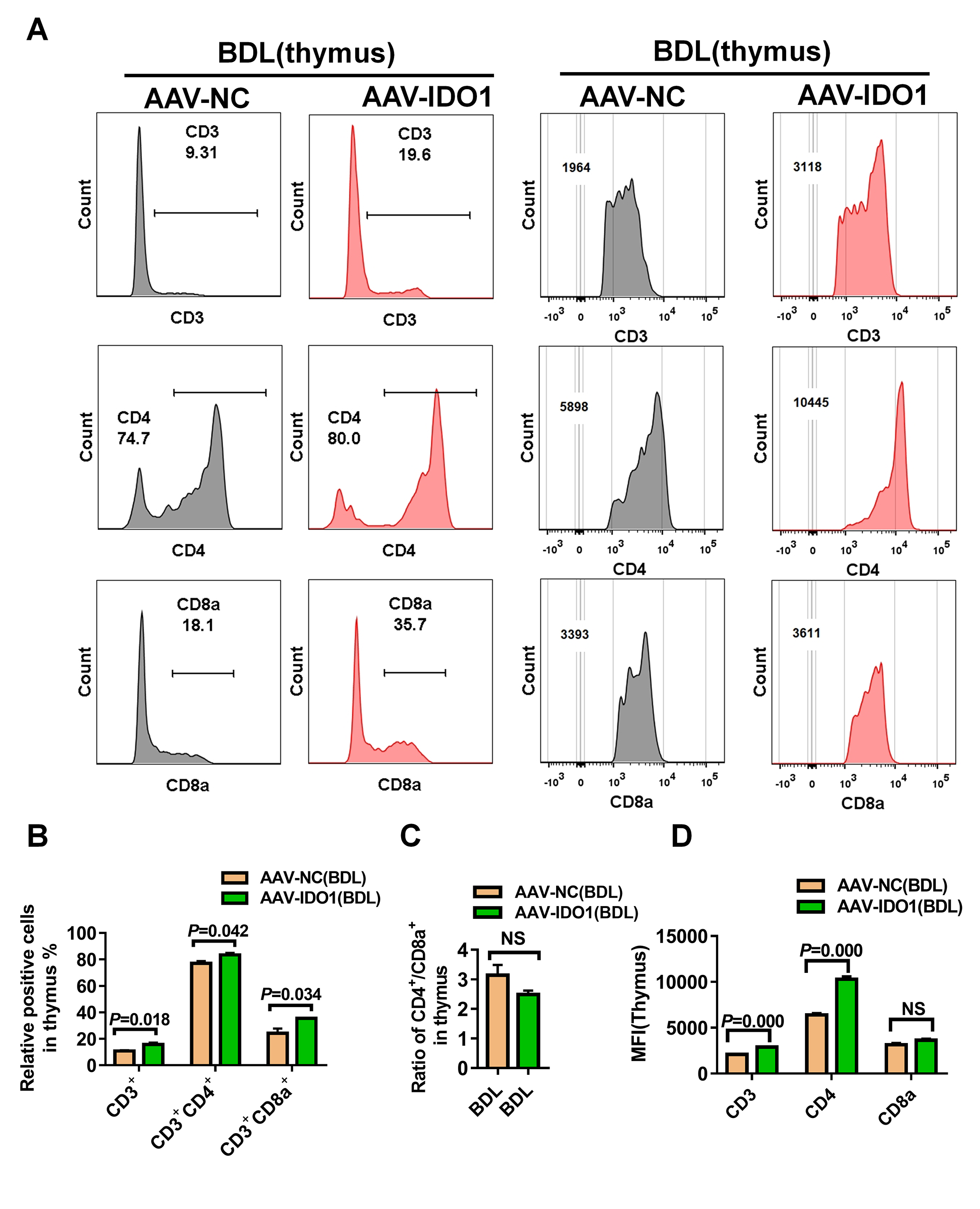

Supplement: Supplementary file 10 — S9: Overexpression of IDO1 suppressed the proliferation rate of thymic T cells during liver fibrosis induced by BDL. [file 41419_2020_3277_MOESM10_ESM.png]
